# Supplementary material for: Identifying DNase I hypersensitive sites as driver distal regulatory elements in breast cancer
Source: Nat Commun. 2017 Sep 5;8:436. doi: 10.1038/s41467-017-00100-x (PMC5585396; doi:10.1038/s41467-017-00100-x)
Supplement: Supplementary file 1 — Supplementary Information [file 41467_2017_100_MOESM1_ESM.pdf]

Type of file: pdf
Size of file: 0 KB
Title of file for HTML: Supplementary Information
Description: Supplementary Figures, Supplementary Tables and Supplementary References.

Type of file: XLSX
Size of file: 0 KB
Title of file for HTML: Supplementary Data 1
Description: All mutated loci in the 47 TCGA discovery samples.

Type of file: XLSX
Size of file: 0 KB
Title of file for HTML: Supplementary Data 2
Description: The number and type of mutations for all 3,147 genes mutated in the 47 discovery samples.

Type of file: XLSX
Size of file: 0 KB
Title of file for HTML: Supplementary Data 3
Description: Enrichment p-values for each mutated DHSs.

Type of file: XLSX
Size of file: 0 KB
Title of file for HTML: Supplementary Data 4
Description: The clusters created to determine which DHSs are mutated at higher levels than expected.

Type of file: XLSX
Size of file: 0 KB
Title of file for HTML: Supplementary Data 5
Description: The sequencing quality for all the DHSs passing Filter 1. 41 DHSs were excluded from further analyses because of poor sequencing quality.

Type of file: XLSX
Size of file: 0 KB
Title of file for HTML: Supplementary Data 6
Description: The associations between each mutated DHS and the expression levels of its target genes.

Type of file: XLSX
Size of file: 0 KB
Title of file for HTML: Supplementary Data 7
Description: All mutated loci in the 50 TCGA replication samples.

Type of file: XLSX
Size of file: 0 KB

Title of file for HTML: Supplementary Data 8
Description: All mutations detected in 12 known cancer genes in the replication samples.

Type of file: XLSX
Size of file: 0 KB
Title of file for HTML: Supplementary Data 9
Description: All mutations detected in the putative driver DHSs in the prevalence screen.

Type of file: XLSX
Size of file: 0 KB
Title of file for HTML: Supplementary Data 10
Description: The enrichment for mutations in each putative driver DHS in comparison its flanking 100 kb.

Type of file: XLSX
Size of file: 0 KB
Title of file for HTML: Supplementary Data 11
Description: All the mutations in the TERT promoter identified in TCGA.

Type of file: XLSX
Size of file: 0 KB
Title of file for HTML: Supplementary Data 12
Description: The sequencing statistics (including total number of reads, duplicated and mapped reads) for all the 97 breast cancer samples from TCGA.

Type of file: XLSX
Size of file: 0 KB
Title of file for HTML: Supplementary Data 13
Description: The list of all amplicons used for targeted sequencing.

Type of file: XLSX
Size of file: 0 KB
Title of file for HTML: Supplementary Data 14
Description: The raw RNA-seq read counts for all genes within 2 Mb of the putative driver DHSs chr8:579137-581436 and 60 and chr20:62115827-62119284.

Type of file: pdf
Size of file: 0 KB
Title of file for HTML: Peer Review File
Description:

Supplementary Figures

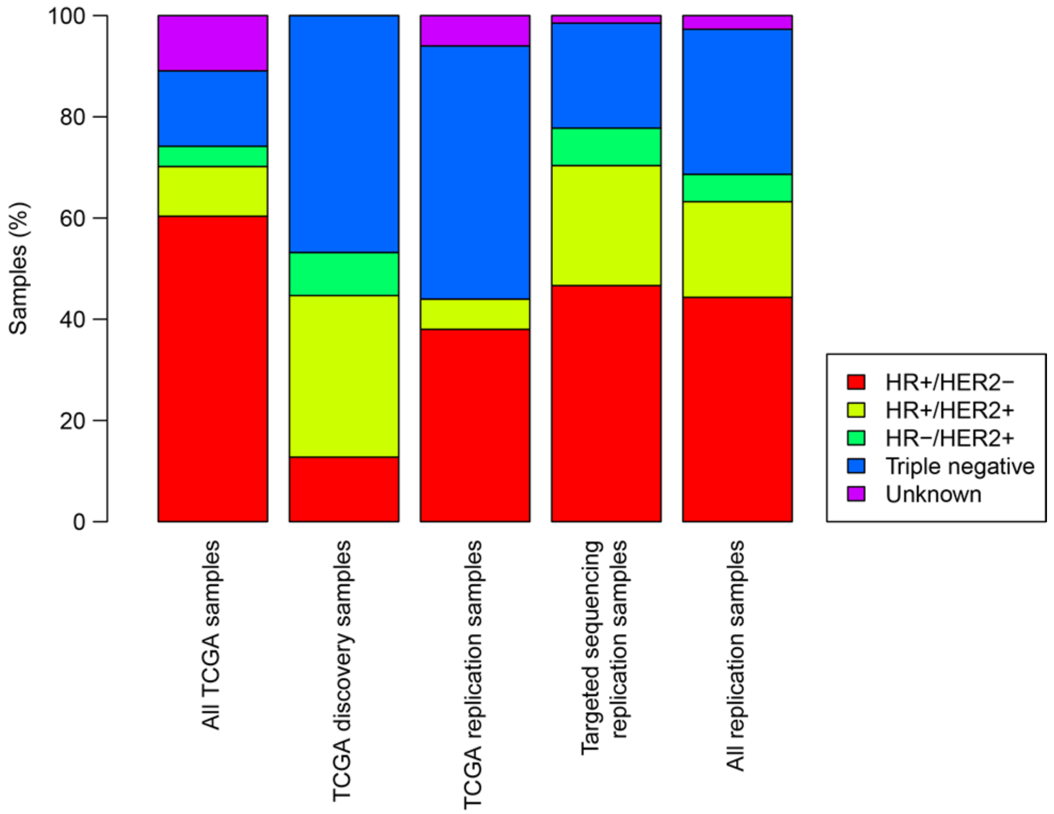

Supplementary Figure 1: Breast cancer clinical phenotypes. ER, PR and HER2 status for the 825 breast cancer samples in the TCGA "All TCGA samples", the 47 TCGA whole-genome discovery samples and 50 TCGA whole-genome replication samples were obtained from the TCGA Data Portal (https://tcga-data.nci.nih.gov/tcga/). The ER, PR and HER2 status for the 135 targeted sequencing samples were obtained from patient records. Clinically relevant breast cancer phenotypes were derived from ER, PR and HER2 statuses as follows[1] : 1) HR+/HER2- contains tumors that are hormone receptor (HR) positive (+), i.e. ER+ and/or PR+, and HER2 negative (-); 2) HR+/HER2+ includes tumors that are ER+ and/or PR+ and HER2+; 3) HR-/HER2+ includes tumors that are ER- and PR- but HER2+; and 4) triple negative includes tumors that are ER-, PR- and HER2-. The distribution of clinical phenotypes across the replication samples "All replication samples" is the combined set of TCGA and targeted sequencing. Not dividing the breast cancer samples into subtypes provided greater sensitivity to detect driver DHSs that are potentially important across all clinically relevant phenotypes as well as in other epithelial cancers[2-4].

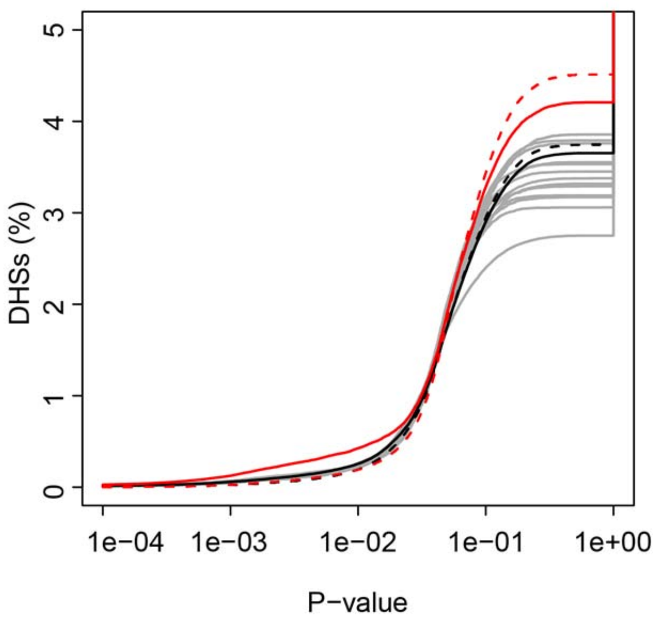

Supplementary Figure 2: Cumulative distributions of test statistic *p*. Cumulative distributions of *p* (Eq. 3) used in Filter 1 for: 1) breast DHSs (red); 2) the 13 control tissues DHSs (one gray line for each); 3) "All control tissues" DHSs represent the union of DHSs that are active in the 13 control tissues (black); 4) and 5) simulated mutations (i.e. expectation under the model of neutral evolution) in breast and control tissues DHSs (dashed), respectively.

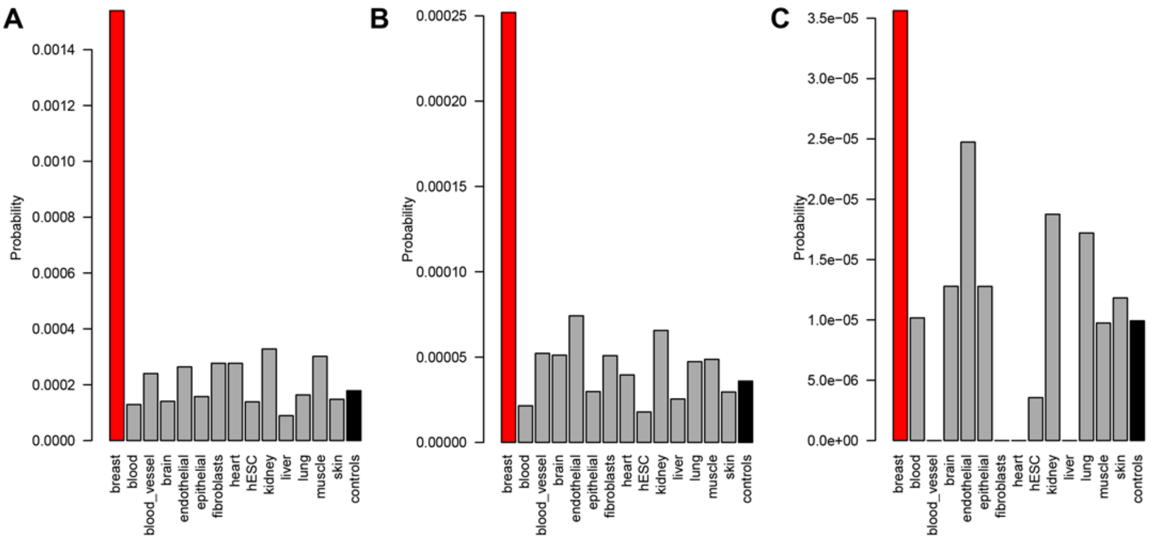

Supplementary Figure 3: Comparisons between breast and control tissues DHSs at Filters 1-3. The fraction of all DHSs passing (**A**) Filter 1 (significantly mutated DHSs), (**B**) Filters 1-3 (DHSs with aberrantly expressed targets), and (**C**) Filters 1-4 (DHSs with mutations in the replication samples) is shown for breast (red), the 13 control tissues (gray) and “All control tissues” combined (black). These plots show that a higher fraction of breast DHSs pass each filter, compared with control tissues DHSs.

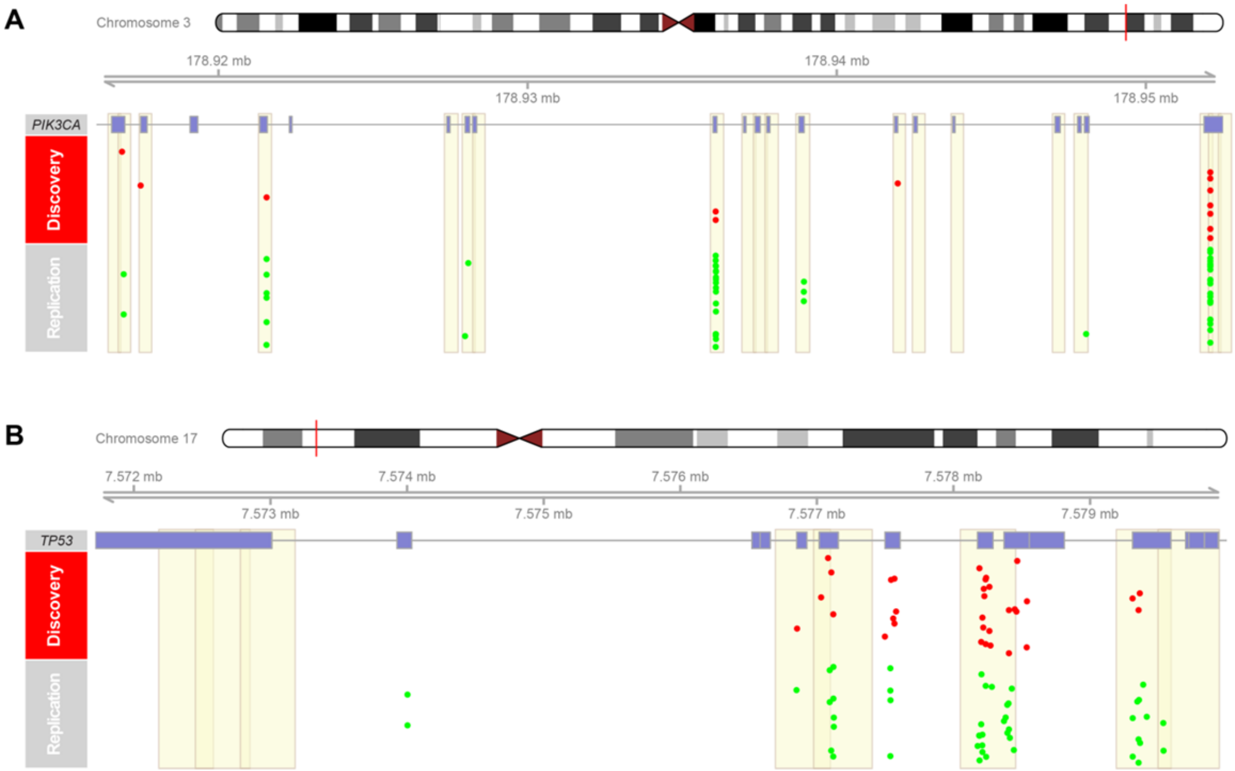

Supplementary Figure 4: Mutation distribution in PIK3CA and TP53 in discovery and replication breast cancer samples. The exons (blue) of *PIK3CA* (**A**) and *TP53* (**B**) covered by the amplicons used for targeted sequencing (yellow). Mutations in the 47 discovery samples are depicted in red and in the 185 replication samples depicted in green. In the replication samples 53 *PIK3CA* mutations (in 45 samples) and 47 *TP53* mutations (in 45 samples) were identified, confirming that they are indeed mutated at high frequency (24.3%). Based on previous studies [3] we expected ~35% of samples to harbor mutations in *TP53* but for technical reasons we did not assay exon 4, which is known to be highly mutated, in the targeted sequencing. For instance exon 4 harbored 7 mutations in the discovery screening (14.8% of all samples).

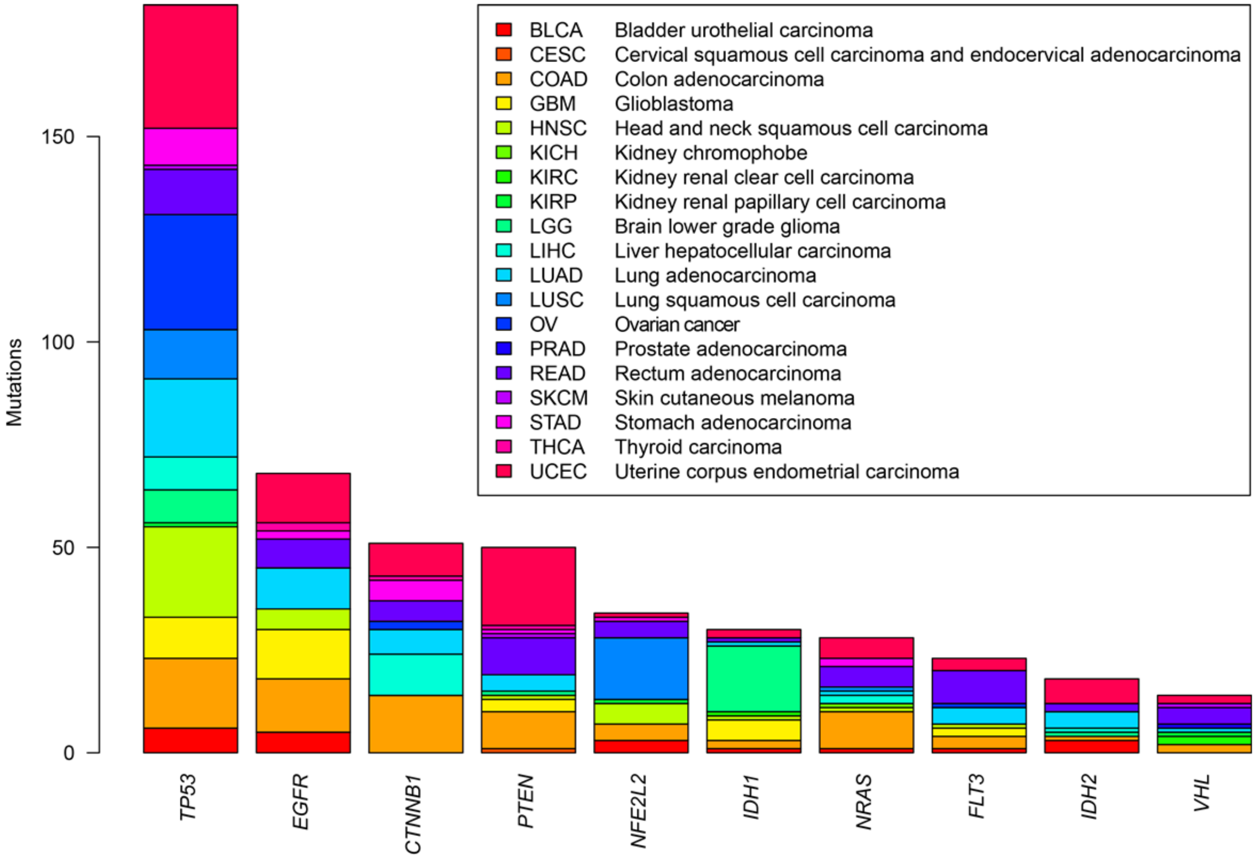

Supplementary Figure 5: Mutation analysis by cancer type of ten genes found as significantly mutated in a recent TCGA pan-cancer analysis. The ten genes used in this analysis were retrieved from Tamborero *et al.* [5]. Somatic mutations were detected in 1,097 samples from 19 tumor types. The number of samples of each cancer type is shown in Figure 3B. *TP53* is the most mutated gene (182 mutations in 147 samples, 13.4% of all tumors). These data show that GTFuse can be used to extract sequences of interest from tumor-normal pairs in CGHub [6] to detect mutations.

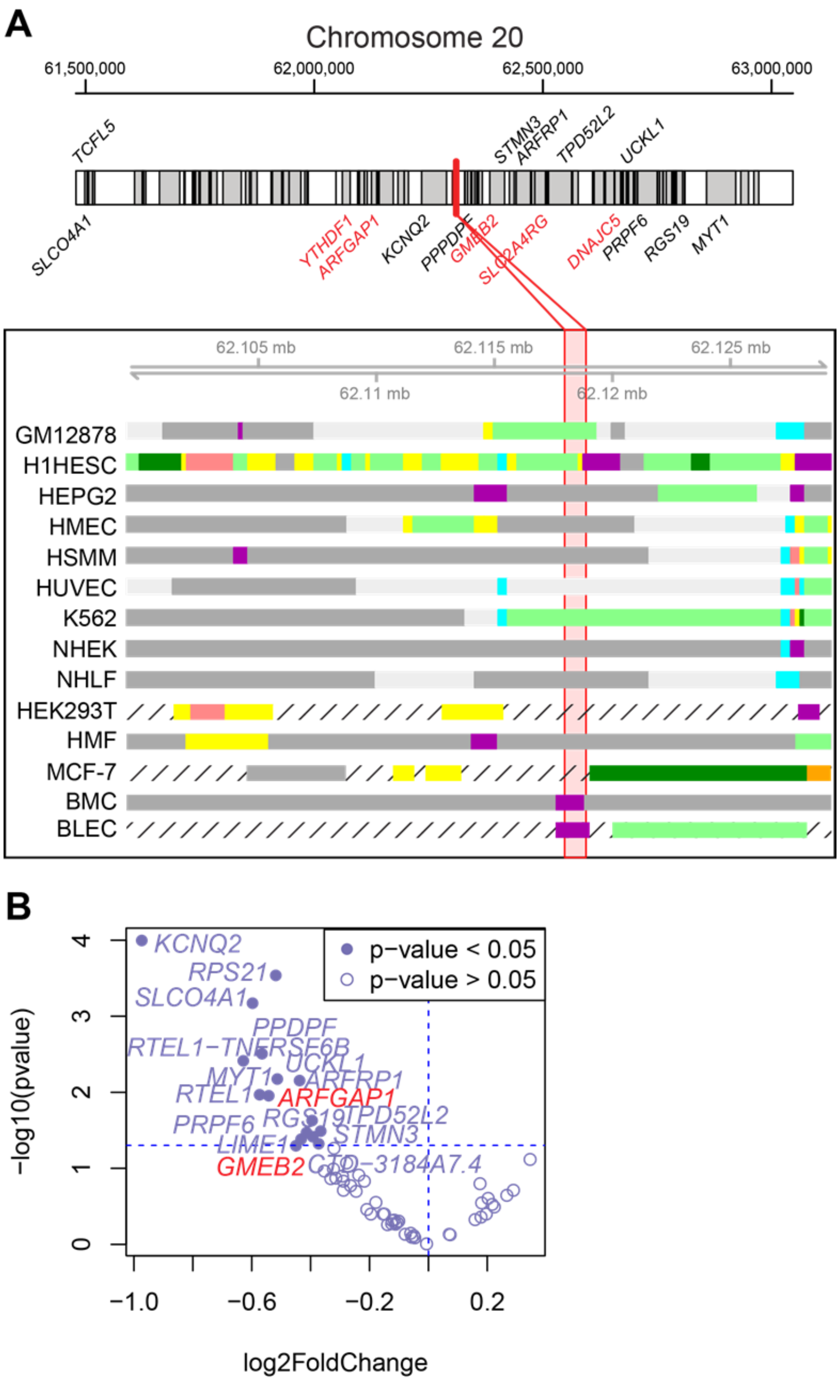

Supplementary Figure 6: Deletion of putative driver DHS chr20:62115827-62119284 results in aberrant expression of nearby genes. (**A**) The relative positions of the deleted putative driver DHS chr20:62115827-62119284 (red) and the neighboring genes expressed in HEK293T are shown. Genes with altered expression associated with mutations and known interactions with the driver DHS in Filter 3 (Target genes) are indicated (red). The chromatin states in 14 cell lines associated with the deleted region are shown. (**B**) Volcano plot showing log2 fold change and p-value of gene expression differences (genes within 1 Mb of the putative driver DHS chr20:62115827-62119284) between cells with deleted putative driver DHS and cells treated with empty vector. Target genes with significantly altered expression between the deleted and empty vector experiments are shown in red.

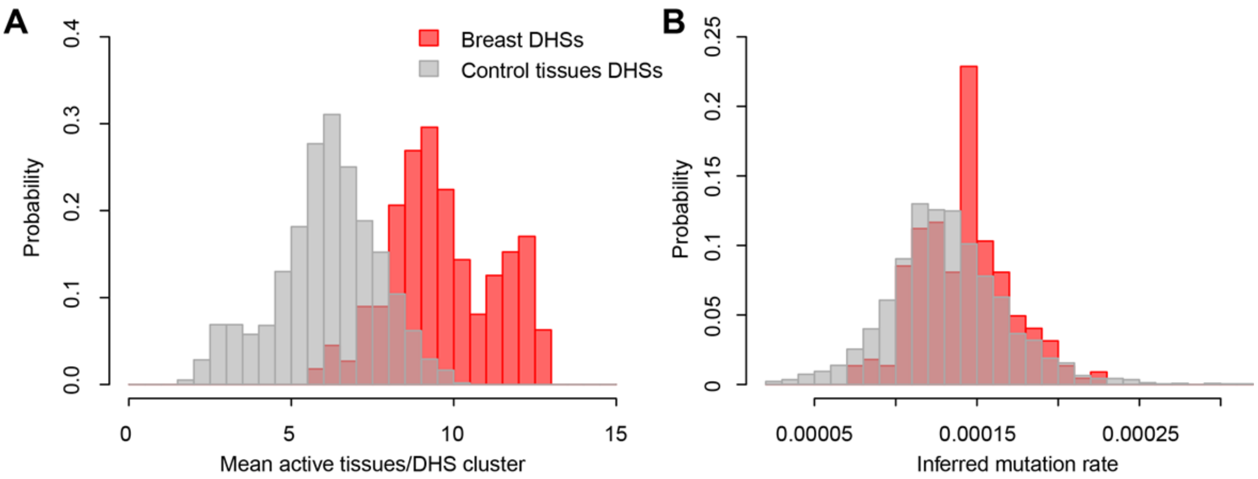

Supplementary Figure 7: Comparison of the number of tissues in which each DHS is active and $\hat{\mu}$ between breast and control tissues DHSs. (**A**) Histogram showing the distribution of the number of tissues in which each DHS is active, averaged by cluster. The plot shows that breast DHSs are expressed in significantly more tissues than DHSs active in control tissues (p-value = $2.27\ 10^{-108}$, Wilcoxon test). This is expected because we removed DHSs in the control tissues that overlap with breast DHSs. Therefore, constitutive DHSs are only included within the list of breast DHSs. The number of tissues in which each DHS is active was calculated by merging all DHSs from breast and all DHSs in the 13 control tissues, using the *mergeBed –c 4 –o count_distinct* function in BedTools: the option –c 4 refers to the BED files used as input, where the fourth column represents the tissue name; the option *–o count_distinct* specifies that the output will include the counts of the number of distinct tissues associated with each set of overlapping DHSs. (**B**) Histogram showing the distribution of the inferred mutation probability for each cluster of DHSs. These plots show that, as expected, breast DHSs are active in a significantly higher number of tissues than control tissues DHSs, but this bias does not affect the inferred mutation rates.

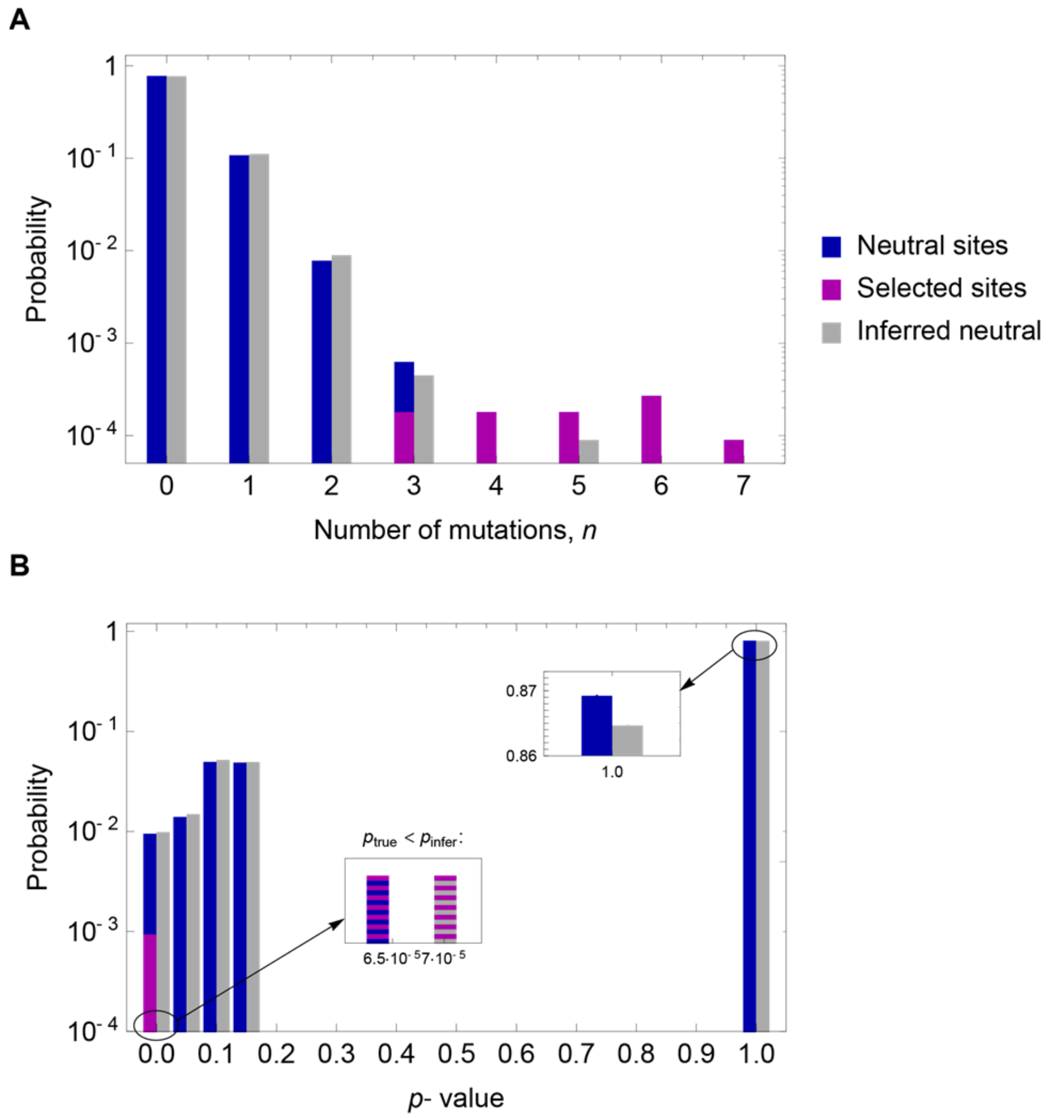

Supplementary Figure 8: The presence of driver DHSs in a cluster leads to overestimation of the inferred mutation probability and a conservative test statistic *p*. (**A**) Shown is a simulated distribution of mutation counts n, each drawn from a Poisson distribution with varying parameter $\lambda = \mu L$. 0.1% of all sites were simulated at a higher mutation probability μ_s than that of neutral sites, μ. The resulting distribution $P(n)$ is shown in magenta/blue. Inference of μ according to Eq. 2 in the presence of selected sites leads to an overestimation of the true neutral mutation probability in the cluster. The resulting inferred neutral distribution is given in gray. (**B**) Histogram of p (Eq. 3) corresponding to the counts in (**A**), based on the inferred neutral mutation probability $\hat{\mu}$ (bin width 0.05). The observed distribution $P(n)$ of neutral and selected counts in (**A**) has a higher variance, leading to an excess at small p as well as for $p = 1$ relative to the expected distribution (top right inset). Importantly, values p of selected sites are overestimated with $\hat{\mu}$ and hence conservative. This can be seen in the lower inset, showing the true and inferred average values $\bar{p}_{\mu}$ (magenta/blue bar) and $\bar{p}_{\hat{\mu}}$ (magenta/gray bar), respectively, for sites under selection. This figure shows that the presence of mutations in driver DHSs creates a bias in the inference of the mutation probability. This bias results in the overestimation of $\hat{\mu}$, therefore the calculation of p for Filter 1 is conservative.

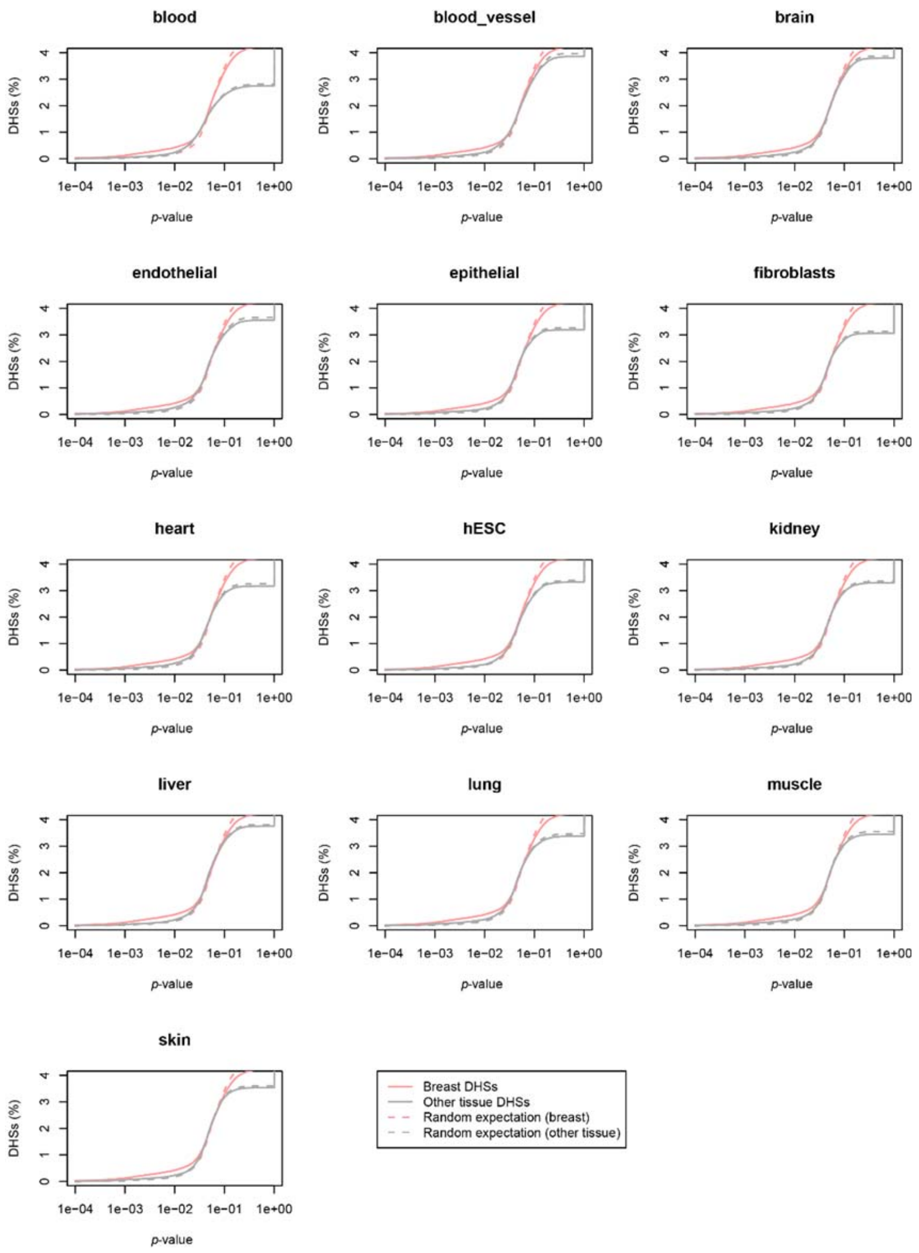

Supplementary Figure 9: Comparison of distributions of test statistic p for observed and simulated DHS mutations. Cumulative distributions of the test statistic *p* for: 1) breast DHSs; 2) DHSs that are active in control tissues; 3) simulated mutations (random expectation), corresponding to the model of neutral evolution, in breast DHSs; and 4) simulated mutations (random expectation) in control tissues DHSs. These data show that for all control tissues DHSs, the difference between the observed and simulated *p* distributions are smaller than what is observed for breast DHSs.

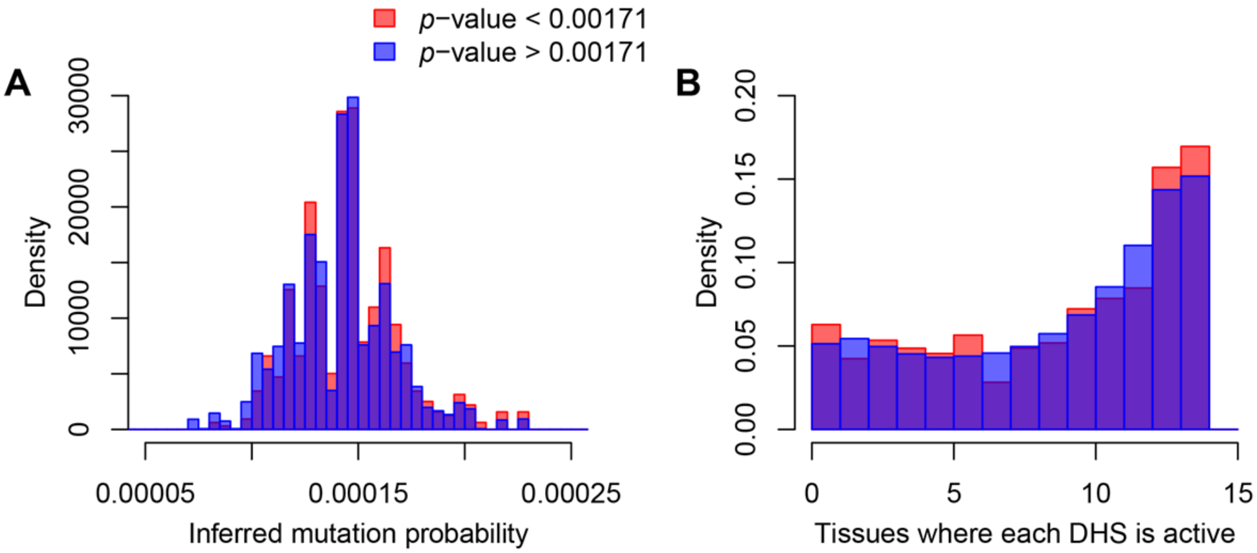

Supplementary Figure 10: Clusters with significantly mutated breast DHSs and clusters without significantly mutated breast DHSs do not differ in terms of inferred mutation rate and number of tissues in which a DHS is active. (**A**) Histogram of the inferred mutation probability $\hat{\mu}$ (Eq. 2) from all 223 clusters of breast DHSs (blue) and of the clusters from which the 637 significantly mutated DHSs with $p < 0.00171$ originate (red). This shows that the significantly mutated breast DHSs we use to determine driver loci form an ensemble that has no severe bias of the background mutation probability. (**B**) Histogram of the number of tissues the full set of 334,781 breast DHSs are active in (blue) and only for the significantly mutated DHSs (red). These plots show that the calculation of p for Filter 1 does not introduce any bias in terms of inferred mutation rate $\hat{\mu}$ and number of tissues in which a breast DHSs is active.

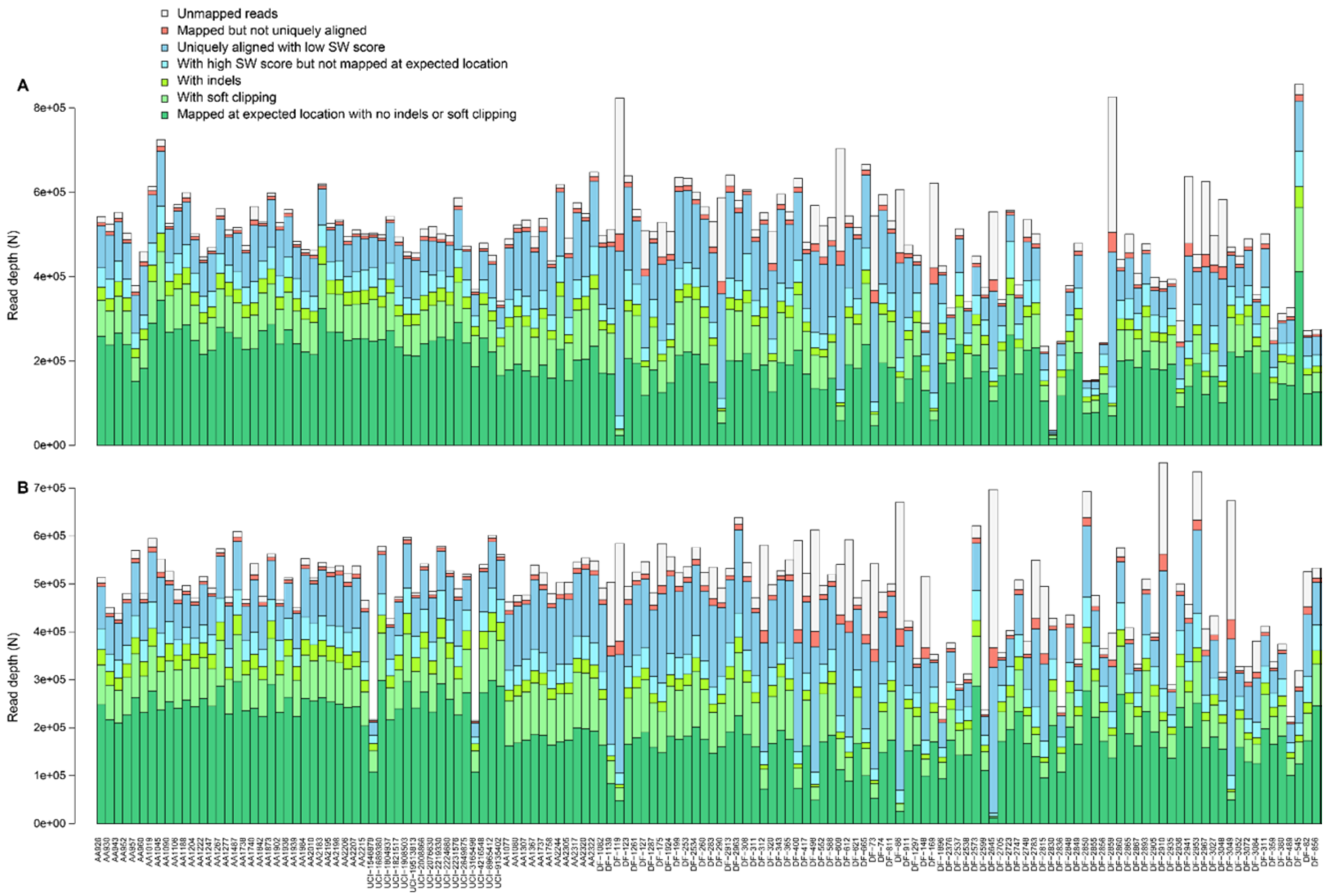

Supplementary Figure 11: Mapping results of targeted sequencing data of 135 matched tumor and normal samples. Tumor samples are shown in (**A**) matched normal samples in (**B**). Alignment statistics were generated using Mutascope[7]. Only reads that were mapped at the expected location with no indels or soft clipping were used for variant calling. With the exception of two tumor (DF−119 and DF−2830) and two normal samples (DF−86 and DF−2645) all matched pairs had data of sufficient quality (average 450X coverage: range 130 to 688) to call mutations at 5% allele frequency in the tumor.

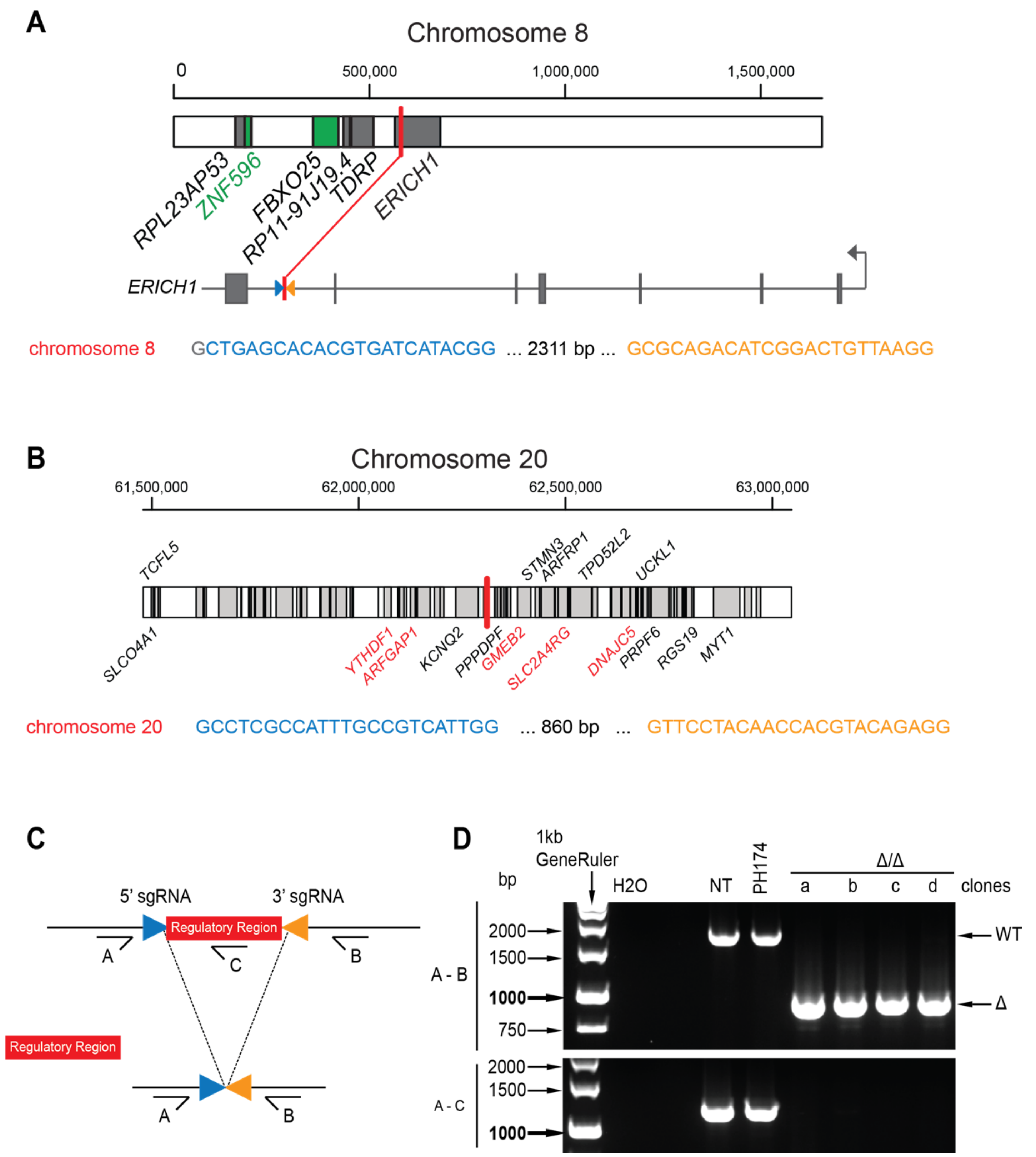

Supplementary Figure 12: Experimental design for the deletion of putative driver DHSs chr8:579137-581436 and chr20:62115827-62119284. (**A**, **B**) Genomic intervals harboring the two putative driver DHSs that were targeted for deletion by CRISPR are shown: chr8:579137-581436 and chr20:62115827-62119284. The deleted DHS interval is shown in red. Target genes of the putative driver DHSs in TCGA breast tumors are shown in green (downregulated) or red (overexpressed, Supplementary Data 5). Primers used by CRISPR are shown (forward in blue; reverse in orange). (**C**)

Schematic of PCR design to assess whether the DHS sequences are deleted. (**D**) Example PCR showing detection of homozygous deletions. The top panel shows PCR results using primers A and B for HEK293T cells with no treatment (NT), with empty vector (PH174), and four deleted clones. The bottom panel is with primers **A** and **C** showing that the PCR amplicon is only amplified in lines for which the putative driver DHS has not been deleted. The putative driver DHS chr8:579137-581436 is located within an *ERICH1* intron it is greater than 30 kb from splice sites, therefore its deletion and that would not be expected to impact the correct splicing of *ERICH1* and we verified that this is indeed the case by examining the RNA-seq data.

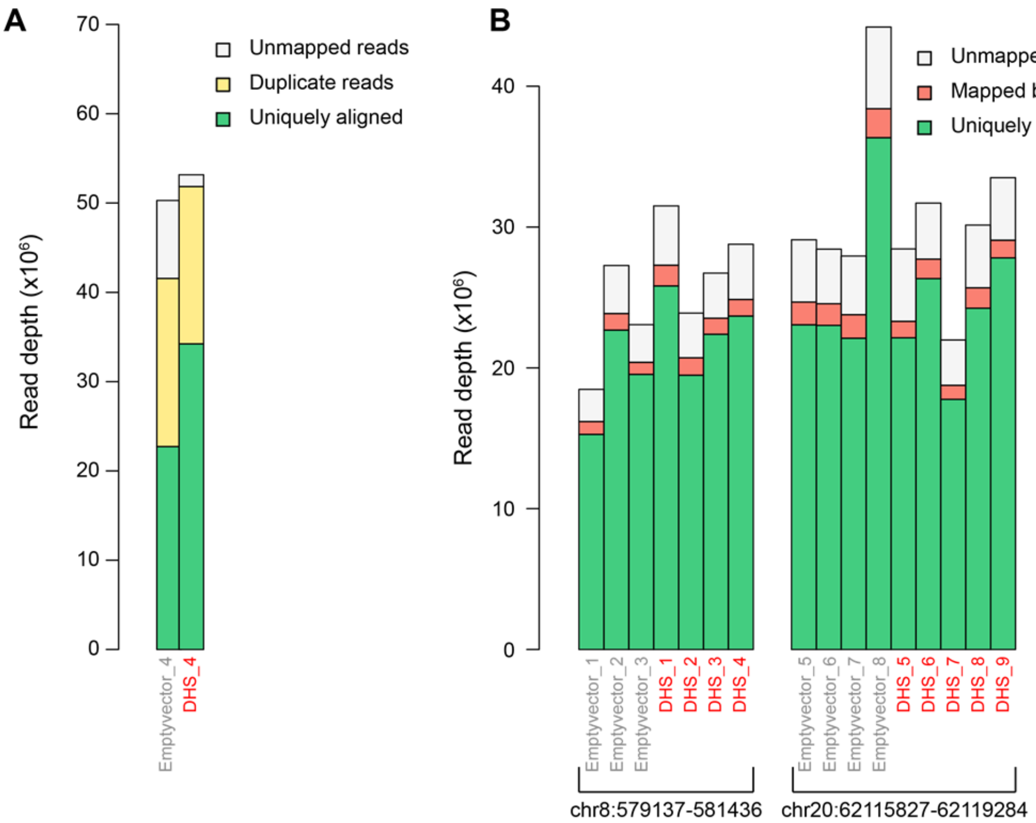

Supplementary Figure 13: Mapping results of ATAC-seq and RNA-seq experiments. Shown are the distributions of sequence reads for (**A**) the two ATAC-seq samples and (**B**) the 16 RNA-seq samples (nine for the analysis of the putative driver DHS on chromosome 20 and seven for the putative driver DHS on chromosome 8). Only uniquely aligned reads (in green) were used to determine chromatin remodeling and differential gene expression. Overall, read depth in all sequenced samples was high (>15 million reads). For the analysis of chr8:579137-581436, three technical replicates for empty vector a four with deleted DHS were used. For chr20:62115827-62119284, four technical replicates with empty vector and five with deleted DHS were used.

Supplementary Tables

Supplementary Table 1: TCGA breast cancer samples with whole genome sequencing data

TCGA ID	Use	Gender	Age at Initial Diagnosis	ER Status	PR Status	HER2 Status	Stage	Clinically relevant phenotype	Subtype
TCGA-A1-A0SM	Discovery	MALE	77	Positive	Negative	Positive	IIA	HR+/HER2+	Luminal B
TCGA-A2-A04P	Discovery	FEMALE	36	Negative	Negative	Negative	IIIC	Triple negative	Basal-like
TCGA-A2-A04Q	Discovery	FEMALE	48	Negative	Negative	Negative	IA	Triple negative	Basal-like
TCGA-A2-A04T	Discovery	FEMALE	62	Negative	Negative	Negative	IIA	Triple negative	Basal-like
TCGA-A2-A04X	Discovery	FEMALE	34	Positive	Positive	Positive	IIA	HR+/HER2+	Luminal B
TCGA-A2-A0CM	Discovery	FEMALE	40	Negative	Negative	Negative	IIA	Triple negative	Basal-like
TCGA-A2-A0D0	Discovery	FEMALE	60	Negative	Negative	Negative	IIA	Triple negative	Basal-like
TCGA-A2-A0D1	Discovery	FEMALE	76	Negative	Negative	Positive	IIA	HR-/HER2+	HER2-enriched
TCGA-A2-A0D2	Discovery	FEMALE	45	Negative	Negative	Negative	IIB	Triple negative	Basal-like
TCGA-A2-A0D4	Replication	FEMALE	37	Positive	Positive	Negative	IIB	HR+/HER2-	Luminal A
TCGA-A2-A0EY	Discovery	FEMALE	62	Positive	Negative	Positive	IIB	HR+/HER2+	Luminal B
TCGA-A2-A0YG	Discovery	FEMALE	63	Positive	Positive	Positive	IIIC	HR+/HER2+	Luminal B
TCGA-A2-A259	Replication	FEMALE	70	Positive	Positive	Negative	IA	HR+/HER2-	Luminal A
TCGA-A2-A25B	Replication	FEMALE	39	Positive	Positive	Negative	IIB	HR+/HER2-	Luminal A
TCGA-A2-A3XX	Replication	FEMALE	49	Negative	Negative	Negative	IIA	Triple negative	Basal-like
TCGA-A2-A3Y0	Replication	FEMALE	57	Positive	Negative	Negative	IIB	HR+/HER2-	Luminal A
TCGA-A7-A0CE	Discovery	FEMALE	57	Negative	Negative	Negative	IIA	Triple negative	Basal-like
TCGA-A7-A13D	Replication	FEMALE	46	Negative	Positive	Negative	IIA	HR+/HER2-	Luminal B
TCGA-A7-A26G	Replication	FEMALE	50	Negative	Negative	Negative	IIA	Triple negative	Basal-like
TCGA-A8-A075	Replication	FEMALE	42	Positive	Positive	Negative	IIB	HR+/HER2-	Luminal A
TCGA-A8-A07B	Discovery	FEMALE	69	Positive	Positive	Positive	IIA	HR+/HER2+	Luminal B
TCGA-A8-A07I	Discovery	FEMALE	69	Positive	Negative	Positive	IIIA	HR+/HER2+	Luminal B
TCGA-A8-A08B	Replication	FEMALE	52	Positive	Negative	Positive	IIA	HR+/HER2+	Luminal B
TCGA-A8-A08L	Discovery	FEMALE	89	Positive	Negative	Negative	IIIA	HR+/HER2-	Luminal A
TCGA-A8-A08S	Discovery	FEMALE	71	Positive	Positive	Positive	IIA	HR+/HER2+	Luminal B
TCGA-A8-A092	Discovery	FEMALE	48	Positive	Positive	Negative	IIIA	HR+/HER2-	Luminal A
TCGA-A8-A094	Discovery	FEMALE	75	Positive	Negative	Negative	IIA	HR+/HER2-	Luminal A
TCGA-A8-A09I	Discovery	FEMALE	84	Positive	Positive	Positive	IIA	HR+/HER2+	Luminal B
TCGA-A8-A09X	Discovery	FEMALE	62	Negative	Negative	Negative	IIIC	Triple negative	Basal-like
TCGA-AC-A2BK	Replication	FEMALE	78	Negative	Negative	Negative	IIIA	Triple negative	Basal-like
TCGA-AN-A04D	Replication	FEMALE	58	Negative	Negative	Negative	IIB	Triple negative	Basal-like
TCGA-AN-A0AT	Replication	FEMALE	62	Negative	Negative	Negative	IIA	Triple negative	Basal-like
TCGA-AN-A0G0	Replication	FEMALE	56	Negative	Negative	Negative	IIA	Triple negative	Basal-like
TCGA-AN-A0XR	Replication	FEMALE	55	Positive	Negative	Negative	IIIA	HR+/HER2-	Luminal A
TCGA-AO-A03L	Discovery	FEMALE	34	Positive	Positive	Negative	IIIA	HR+/HER2-	Luminal A
TCGA-AO-A03N	Discovery	FEMALE	59	Positive	Positive	Negative	IIB	HR+/HER2-	Luminal A

TCGA-AO-A0J2	Discovery	FEMALE	41	Negative	Negative	Negative	IA	Triple negative	Basal-like
TCGA-AO-A0J4	Discovery	FEMALE	41	Negative	Negative	Negative	IA	Triple negative	Basal-like
TCGA-AO-A0J6	Discovery	FEMALE	61	Negative	Negative	Negative	IIA	Triple negative	Basal-like
TCGA-AO-A0JM	Replication	FEMALE	40	Positive	Positive	Positive	IIB	HR+/HER2+	Luminal B
TCGA-AO-A124	Replication	FEMALE	38	Negative	Negative	Negative	IIA	Triple negative	Basal-like
TCGA-AO-A12H	Replication	FEMALE	69	Positive	Positive	Negative	IIA	HR+/HER2-	Luminal A
TCGA-AQ-A04J	Discovery	FEMALE	45	Negative	Negative	Negative	IIA	Triple negative	Basal-like
TCGA-AR-A0TX	Discovery	FEMALE	64	Positive	Positive	Positive	II	HR+/HER2+	Luminal B
TCGA-AR-A1AY	Replication	FEMALE	65	Negative	Negative	Negative	I	Triple negative	Basal-like
TCGA-AR-A24Z	Replication	FEMALE	57	Positive	Positive	Negative	II	HR+/HER2-	Luminal A
TCGA-AR-A256	Replication	FEMALE	45	Negative	Negative	Negative	II	Triple negative	Basal-like
TCGA-AR-A2LK	Replication	FEMALE	62	Positive	Positive	NA	III	Unknown	NA
TCGA-B6-A0I1	Replication	FEMALE	93	Negative	Negative	NA	IIA	Unknown	NA
TCGA-B6-A0I2	Discovery	FEMALE	45	NA	NA	Negative	IA	Triple negative	Basal-like
TCGA-B6-A0I6	Discovery	FEMALE	49	Negative	Negative	NA	IIA	Triple negative	Basal-like
TCGA-B6-A0IJ	Discovery	FEMALE	42	Positive	Positive	Negative	IIB	HR+/HER2-	Basal-like
TCGA-B6-A0IQ	Discovery	FEMALE	40	Negative	Negative	Negative	IIIA	Triple negative	Basal-like
TCGA-B6-A0RE	Discovery	FEMALE	61	Negative	Negative	Negative	NA	Triple negative	Basal-like
TCGA-B6-A0RT	Discovery	FEMALE	39	Negative	Negative	Negative	IIIA	Triple negative	Basal-like
TCGA-B6-A0RU	Discovery	FEMALE	49	Negative	Negative	Negative	IA	Triple negative	Basal-like
TCGA-B6-A0WX	Replication	FEMALE	40	Negative	Negative	Negative	IIIA	Triple negative	Basal-like
TCGA-BH-A0AV	Replication	FEMALE	52	Negative	Negative	Negative	I	Triple negative	Basal-like
TCGA-BH-A0B3	Discovery	FEMALE	53	Negative	Negative	Negative	IIB	Triple negative	Basal-like
TCGA-BH-A0B9	Discovery	FEMALE	44	Negative	Negative	Negative	IA	Triple negative	Basal-like
TCGA-BH-A0BW	Replication	FEMALE	71	Negative	Negative	Negative	I	Triple negative	Basal-like
TCGA-BH-A0DG	Replication	FEMALE	30	Positive	Negative	Negative	IIA	HR+/HER2-	Luminal A
TCGA-BH-A0DT	Replication	FEMALE	41	Positive	Positive	Negative	IIA	HR+/HER2-	Luminal A
TCGA-BH-A0E0	Discovery	FEMALE	38	Negative	Negative	Negative	IIIC	Triple negative	Basal-like
TCGA-BH-A0EA	Replication	FEMALE	72	Positive	Positive	Negative	IIA	HR+/HER2-	Luminal A
TCGA-BH-A0H0	Replication	FEMALE	69	Positive	Positive	Negative	IA	HR+/HER2-	Luminal B
TCGA-BH-A0H6	Replication	FEMALE	82	Positive	Positive	Negative	IA	HR+/HER2-	Luminal A
TCGA-BH-A0WA	Discovery	FEMALE	82	Negative	Negative	Negative	IA	Triple negative	Basal-like
TCGA-BH-A18R	Discovery	FEMALE	50	Positive	Negative	Positive	IB	HR+/HER2+	Luminal B
TCGA-BH-A18U	Discovery	FEMALE	72	Positive	Positive	Positive	IIIA	HR+/HER2+	Luminal B
TCGA-BH-A1FC	Replication	FEMALE	78	Negative	Negative	Negative	IIA	Triple negative	Basal-like
TCGA-C8-A12L	Discovery	FEMALE	67	Negative	Negative	Positive	IIA	HR-/HER2+	HER2-enriched
TCGA-C8-A12Q	Discovery	FEMALE	78	Negative	Negative	Positive	IIIA	HR-/HER2+	HER2-enriched

TCGA-C8-A130	Discovery	FEMALE	52	Positive	Positive	Positive	IIIA	HR+/HER2+	Luminal B
TCGA-D8-A27F	Replication	FEMALE	40	Negative	Negative	Negative	IIA	Triple negative	Basal-like
TCGA-D8-A27H	Replication	FEMALE	72	Negative	Negative	Negative	IIA	Triple negative	Basal-like
TCGA-E2-A109	Replication	FEMALE	64	Positive	Negative	Negative	IIA	HR+/HER2-	Luminal A
TCGA-E2-A14P	Discovery	FEMALE	79	Negative	Negative	Positive	IIIC	HR-/HER2+	HER2-enriched
TCGA-E2-A14X	Replication	FEMALE	55	Negative	Negative	Negative	IIIA	Triple negative	Basal-like
TCGA-E2-A152	Discovery	FEMALE	56	Positive	Negative	Positive	I	HR+/HER2+	Luminal B
TCGA-E2-A156	Replication	FEMALE	61	Positive	Positive	Negative	I	HR+/HER2-	Luminal A
TCGA-E2-A15E	Discovery	FEMALE	40	Positive	Positive	Positive	IIA	HR+/HER2+	Luminal B
TCGA-E2-A15H	Discovery	FEMALE	38	Positive	Positive	Positive	IIA	HR+/HER2+	Luminal B
TCGA-E2-A15K	Replication	FEMALE	58	Positive	Positive	Negative	IIA	HR+/HER2-	Luminal B
TCGA-E2-A1LG	Replication	FEMALE	50	Negative	Negative	Negative	IIA	Triple negative	Basal-like
TCGA-E2-A1LK	Replication	FEMALE	84	Negative	Negative	Negative	IIIC	Triple negative	Basal-like
TCGA-E2-A1LL	Replication	FEMALE	73	Negative	Negative	Negative	IIIA	Triple negative	Basal-like
TCGA-E9-A1NH	Replication	FEMALE	71	Positive	Positive	Negative	IIB	HR+/HER2-	Luminal A
TCGA-EW-A1J5	Replication	FEMALE	59	Positive	Positive	Negative	IIB	HR+/HER2-	Luminal A
TCGA-EW-A1P8	Replication	FEMALE	58	Negative	Negative	Negative	IIIC	Triple negative	Basal-like
TCGA-EW-A1PB	Replication	FEMALE	70	Negative	Negative	Negative	IIIA	Triple negative	Basal-like
TCGA-EW-A1PC	Replication	FEMALE	66	Positive	Positive	Positive	IIB	HR+/HER2+	Luminal B
TCGA-EW-A1PH	Replication	FEMALE	52	Negative	Negative	Negative	IIA	Triple negative	Basal-like
TCGA-EW-A3U0	Replication	FEMALE	61	Negative	Negative	Negative	IIIA	Triple negative	Basal-like
TCGA-GI-A2C9	Replication	FEMALE	59	Negative	Negative	Negative	IIB	Triple negative	Basal-like
TCGA-GM-A2DF	Replication	FEMALE	53	Negative	Negative	Negative	NA	Triple negative	Basal-like
TCGA-GM-A3XL	Replication	FEMALE	49	Negative	Negative	NA	IIA	Unknown	NA

Breast cancer samples in TCGA with WGS were divided into two groups: “discovery” and “replication”. Data shown in the table shows clinical information including subtypes derived from the TCGA breast cancer paper[3].

Supplementary Table 2: Number of somatic mutations in the 47 discovery samples

Barcode	Mutations	Outside repeats	Not in dbSNP	Outside repeats and Not in dbSNP	Mutations in all coding regions	Mutations in 12 driver genes
TCGA-A1-A0SM	3,241	1,229	2,447	1,043	25	
TCGA-A2-A04P	11,651	5,333	10,677	5,070	91	*PIK3CA, TP53*
TCGA-A2-A04Q	4,424	1,466	3,129	1,221	10	
TCGA-A2-A04T	9,025	4,057	8,143	3,834	75	*PIK3CA, TP53*
TCGA-A2-A04X	5,982	2,294	4,666	2,014	22	
TCGA-A2-A0CM	12,098	4,081	9,815	3,608	47	*TP53*
TCGA-A2-A0D0	9,643	4,262	8,640	3,961	53	*TP53*
TCGA-A2-A0D1	6,082	2,450	4,927	2,170	56	*PIK3CA, TP53*
TCGA-A2-A0D2	9,297	4,111	8,235	3,876	67	
TCGA-A2-A0EY	23,262	10,533	21,532	10,040	149	*PIK3CA, TP53*
TCGA-A2-A0YG	6,767	2,754	5,642	2,462	38	*TP53*
TCGA-A7-A0CE	10,067	4,587	9,087	4,300	102	*TP53*
TCGA-A8-A07B	8,257	3,388	6,996	3,094	52	*PIK3CA, CDH1*
TCGA-A8-A07I	6,656	2,659	5,385	2,333	32	
TCGA-A8-A08L	24,664	11,618	23,278	11,213	162	*PIK3CA*
TCGA-A8-A08S	5,327	2,157	4,292	1,902	41	
TCGA-A8-A092	9,509	3,930	8,371	3,624	74	*PIK3CA, TP53*
TCGA-A8-A094	41,714	19,184	38,654	18,324	139	*TP53*
TCGA-A8-A09I	16,195	7,079	14,146	6,551	101	
TCGA-A8-A09X	6,543	2,791	5,586	2,507	47	*TP53, CDH1*
TCGA-AO-A03L	4,523	1,647	3,420	1,422	23	*PIK3CA, TP53*
TCGA-AO-A03N	11,169	4,892	9,884	4,557	76	*PIK3CA, TP53*
TCGA-AO-A0J2	10,023	4,402	8,907	4,116	52	*TP53*
TCGA-AO-A0J4	9,483	4,064	8,370	3,794	74	
TCGA-AO-A0J6	11,326	5,002	10,114	4,703	91	*TP53*
TCGA-AQ-A04J	8,608	3,253	7,151	2,883	41	*TP53*
TCGA-AR-A0TX	32,160	15,110	30,516	14,608	225	*TP53, MLL3*
TCGA-B6-A0I2	5,866	2,264	4,778	2,003	42	
TCGA-B6-A0I6	13,510	4,950	11,737	4,560	84	
TCGA-B6-A0IJ	15,266	6,567	13,541	6,145	115	*TP53, MLL3*
TCGA-B6-A0IQ	8,422	3,299	7,161	2,998	49	*TP53*
TCGA-B6-A0RE	13,517	6,172	12,432	5,860	82	*TP53*
TCGA-B6-A0RT	5,050	1,500	3,729	1,290	12	
TCGA-B6-A0RU						*TP53*

	6,150	2,445	5,051	2,158	37	
TCGA-BH-A0B3	4,371	1,663	3,653	1,473	33	*TP53*
TCGA-BH-A0B9	4,947	1,801	3,887	1,583	31	*TP53*
TCGA-BH-A0E0	5,025	2,049	4,219	1,875	27	*TP53*
TCGA-BH-A0WA	9,035	4,072	8,199	3,833	74	*TP53*
TCGA-BH-A18R	5,505	2,115	4,344	1,868	19	
TCGA-BH-A18U	12,113	5,423	10,834	5,068	66	*TP53*
TCGA-C8-A12L	8,992	3,839	7,572	3,465	60	*PIK3CA, TP53*
TCGA-C8-A12Q	13,376	6,062	12,018	5,738	75	*TP53*
TCGA-C8-A130	5,691	2,172	4,590	1,941	28	*PIK3CA, TP53*
TCGA-E2-A14P	10,049	4,173	8,743	3,872	58	*TP53*
TCGA-E2-A152	15,756	6,970	14,254	6,535	97	*TP53*
TCGA-E2-A15E	4,792	1,561	3,634	1,334	34	*PIK3CA, TP53*
TCGA-E2-A15H	3,656	1,334	2,678	1,129	28	*GATA3*
Total	488,785	208,764	429,064	193,958	3,016	
Mean	10,400	4,442	9,129	4,127	64	
Standard deviation	7,343	3,510	7,042	3,409	42	

For each discovery sample, the total number of mutations, the number retained after filtering those in repeat elements and the number retained after filtering those in same loci as known SNPs is given. Mutations in coding regions were obtained by intersecting the coordinates of mutations "outside repeats and not overlapping SNPs" with the coordinates of exons in RefSeq. Tumors carrying mutations in the 12 most mutated genes in breast cancer (*TP53, PIK3CA, MAP3K1, MAP2K4, GATA3, MLL3, CDH1, PTEN, PIK3R1, RUNX1, TBX3, CTCF*)[3] are indicated.

Supplementary Table 3: Kataegis loci in the 47 discovery samples

Barcode	Chromosome	Start	End	Length	Mutations
TCGA-A2-A04T	20	23,032,942	23,034,704	1,762	8
TCGA-A2-A0D1	7	21,851,455	21,851,755	300	6
TCGA-A2-A0D1	8	37,659,335	37,659,860	525	6
TCGA-A2-A0D1	8	37,700,295	37,700,919	624	10
TCGA-A2-A0D1	8	58,443,184	58,444,071	887	8
TCGA-A2-A0D1	8	72,928,240	72,931,013	2,773	6
TCGA-A2-A0EY	1	16,259,369	16,263,369	4,000	9
TCGA-A2-A0EY	3	112,944,536	112,945,519	983	6
TCGA-A2-A0EY	4	169,678,393	169,682,344	3,951	6
TCGA-A2-A0EY	6	44,400,208	44,407,911	7,703	9
TCGA-A2-A0EY	9	127,213,358	127,217,554	4,196	7
TCGA-A2-A0EY	12	9,252,514	9,255,974	3,460	13
TCGA-A2-A0EY	12	9,262,140	9,266,681	4,541	7
TCGA-A2-A0EY	12	33,903,108	33,903,588	480	6
TCGA-A2-A0EY	14	51,730,888	51,731,888	1,000	7
TCGA-A2-A0EY	22	38,506,441	38,512,084	5,643	6
TCGA-A2-A0YG	1	156,012,150	156,013,281	1,131	7
TCGA-A2-A0YG	17	37,135,494	37,136,758	1,264	10
TCGA-A2-A0YG	17	68,236,999	68,241,345	4,346	10
TCGA-A8-A07B	6	104,702,242	104,705,888	3,646	18
TCGA-A8-A07B	10	11,557,279	11,558,269	990	6
TCGA-A8-A07B	17	38,588,845	38,592,720	3,875	7
TCGA-A8-A07B	17	47,991,548	47,993,890	2,342	9
TCGA-A8-A07I	1	206,691,371	206,691,962	591	7
TCGA-A8-A07I	5	176,920,569	176,921,077	508	6
TCGA-A8-A07I	17	35,348,085	35,348,723	638	7
TCGA-A8-A08L	10	552,529	556,915	4,386	8
TCGA-A8-A08S	17	11,545,905	11,546,728	823	6
TCGA-A8-A092	6	79,100,755	79,105,982	5,227	7
TCGA-A8-A094	11	70,227,346	70,228,031	685	6
TCGA-A8-A094	11	72,021,990	72,025,477	3,487	8
TCGA-A8-A094	14	64,494,033	64,497,798	3,765	7
TCGA-A8-A094	14	64,496,933	64,502,142	5,209	6
TCGA-A8-A09I	4	179,916,674	179,920,696	4,022	7
TCGA-A8-A09I	17	47,575,829	47,576,908	1,079	6
TCGA-A8-A09I	17	50,083,291	50,088,314	5,023	8
TCGA-A8-A09X	17	62,602,674	62,605,074	2,400	6
TCGA-AO-A03L	17	36,045,536	36,046,632	1,096	6
TCGA-AO-A03L	17	62,022,649	62,023,238	589	7
TCGA-AO-A03N	6	24,866,014	24,870,295	4,281	8
TCGA-AO-A03N	14	39,365,583	39,371,162	5,579	8
TCGA-AO-A0J4	4	41,207,566	41,211,336	3,770	8
TCGA-AO-A0J6	4	60,430,427	60,436,313	5,886	6
TCGA-AR-A0TX	3	404,828	406,798	1,970	9
TCGA-AR-A0TX	5	141,129,047	141,133,447	4,400	7
TCGA-AR-A0TX	12	71,950,715	71,952,574	1,859	18
TCGA-AR-A0TX	22	45,290,464	45,292,151	1,687	6
TCGA-AR-A0TX	22	45,327,933	45,333,222	5,289	12
TCGA-B6-A0I2	11	12,021,427	12,029,713	8,286	15

TCGA-B6-A0I2	11	20,470,068	20,474,307	4,239	15
TCGA-B6-A0I2	19	58,034,134	58,035,287	1,153	6
TCGA-B6-A0I2	19	58,050,928	58,052,938	2,010	8
TCGA-B6-A0IJ	5	158,509,045	158,515,725	6,680	21
TCGA-B6-A0IJ	5	159,334,934	159,341,701	6,767	19
TCGA-B6-A0IJ	8	75,835,306	75,838,460	3,154	10
TCGA-B6-A0IQ	X	92,476,864	92,480,247	3,383	13
TCGA-BH-A0E0	11	93,274,832	93,280,476	5,644	10
TCGA-BH-A0WA	3	127,114,492	127,115,577	1,085	10
TCGA-BH-A0WA	5	85,485,660	85,486,578	918	6
TCGA-BH-A18U	7	52,435,879	52,436,584	705	7
TCGA-C8-A12L	1	94,528,520	94,533,145	4,625	15
TCGA-C8-A12Q	11	51,481,124	51,486,972	5,848	11
TCGA-C8-A12Q	22	38,009,864	38,013,218	3,354	6
TCGA-C8-A130	12	93,113,082	93,116,188	3,106	6
TCGA-C8-A130	13	22,848,441	22,849,810	1,369	6
TCGA-C8-A130	13	40,238,558	40,241,564	3,006	10
TCGA-E2-A14P	10	12,678,941	12,680,251	1,310	8
TCGA-E2-A152	17	36,513,811	36,514,825	1,014	9
TCGA-E2-A15E	8	30,282,266	30,286,142	3,876	10

We show the coordinates and number of mutations in each of the 69 kataegis loci found in 29 TCGA discovery samples. Kataegis loci are defined as stretches of at least 6 consecutive mutations with intermutation distances < 1000 bp.

Supplementary Table 4: Collapsing 53 ENCODE cell lines into 13 control tissue types

Cell line	Tissue type
Cd4naivewb11970640	Blood
Gm06990	Blood
Gm12865	Blood
K562	Blood
K562Znfa41c6	Blood
K562Znfp5	Blood
Monocd14ro1746	Blood
Nb4	Blood
Th1	Blood
Th17	Blood
Th1wb33676984	Blood
Th2	Blood
Th2wb54553204	Blood
Tregwb78495824	Blood
Hpaf	Blood vessel
Huvec	Blood vessel
Hah	Brain
Hasp	Brain
M059j	Brain
Nha	Brain
Sknshra	Brain
Hmvecb	Endothelial
Hmvecd	Endothelial
Hmvecdblad	Endothelial
Hmvecf	Endothelial
Hmvech	Endothelial
Hcpe	Epithelial
Hee	Epithelial
Hipe	Epithelial
Hpdlf	Epithelial
Saec	Epithelial
Aoaf	Fibroblasts
Hgf	Fibroblasts
Hvmf	Fibroblasts
Hcf	Heart
Hcfaa	Heart
Hcm	Heart
H7es	hESC
Hrce	Kidney
Hepg2	Liver
A549	Lung
Cd20ro01778	Lung
Hpf	Lung
Nhlf	Lung
Hsmm	Muscle
Lhcnm2	Muscle
Lhcnm2Diff4d	Muscle
Skmc	Muscle
Ag10803	Skin

Hff	Skin
Nhdfad	Skin
Nhdfneo	Skin
Rpmi7951	Skin

For each of the 53 ENCODE cell lines with DHS data used in this study we show the associated control tissue.

Supplementary Table 5: Negative controls

Tissue type	DHSs (N)	DHSs that do not overlap breast DHSs (N)	DHSs not overlapping kataegis or repetitive elements	Mutated DHSs (N)	Clusters	KS test	P-value for FDR = 0.25
Breast	392,977	NA	334,781	14,087	223	0.005	0.001710
Blood	1,137,583	884,950	676,840	18,653	311	0.920	0.000220
Blood vessel	275,756	95,832	81,642	3,150	124	1.000	0.000360
Brain	470,284	234,714	197,273	7,490	172	1.000	0.000240
Endothelial	280,845	121,281	104,059	3,700	146	1.000	0.000390
Epithelial	491,790	234,839	192,507	6,150	240	1.000	0.000290
Fibroblasts	398,166	177,028	147,637	4,522	216	1.000	0.000430
Heart	309,438	101,189	84,276	2,679	151	1.000	0.000440
hESC	450,533	280,868	212,139	7,046	164	1.000	0.000260
Kidney	277,700	106,669	91,378	3,016	122	1.000	0.000470
Liver	156,560	78,584	70,482	2,656	116	1.000	0.000150
Lung	476,844	232,494	192,804	6,523	193	1.000	0.000260
Muscle	443,342	205,343	168,441	5,823	171	1.000	0.000470
Skin	405,710	169,184	141,990	5,020	199	1.000	0.000280
All control tissues	NA	1,363,604	1,064,296	38,882	356	0.870	0.000328

For each of the 13 control tissues we show the number of DHSs, the number of DHSs retained after removing those that overlap breast DHSs, the number of DHSs retained after removing those that overlap kataegis loci and repetitive elements, the total number of mutated DHSs, and the number of clusters. The last row “All control tissues” represents the union of all DHSs in the 13 control tissues. “KS test” represents the p-value from Kolmogorov-Smirnov test to assess the probability with which the observed values of the test statistic p in control tissues stem from the expected distribution simulated under the neutral Poisson model.

Supplementary Table 6: Number of somatic mutations in the 50 TCGA replication samples

Barcode	Mutations	Outside repeats	Not in dbSNP	Outside repeats and Not in dbSNP
TCGA-A2-A0D4	6,149	2,268	5,046	2,007
TCGA-A2-A259	3,951	1,314	2,691	1,047
TCGA-A2-A25B	13,283	5,625	11,747	5,242
TCGA-A2-A3XX	5,476	1,943	4,240	1,687
TCGA-A2-A3Y0	15,936	7,053	14,461	6,657
TCGA-A7-A13D	10,821	4,556	9,464	4,204
TCGA-A7-A26G	7,708	2,907	6,323	2,598
TCGA-A8-A075	9,637	4,154	8,459	3,905
TCGA-A8-A08B	6,146	2,239	4,922	1,963
TCGA-AC-A2BK	11,473	4,851	10,162	4,494
TCGA-AN-A04D	9,789	4,129	8,481	3,857
TCGA-AN-A0AT	9,975	4,273	8,887	4,021
TCGA-AN-A0G0	5,959	2,235	4,592	1,960
TCGA-AN-A0XR	5,153	1,893	3,895	1,628
TCGA-AO-A0JM	5,548	2,005	4,365	1,778
TCGA-AO-A124	17,191	7,890	15,822	7,494
TCGA-AO-A12H	4,749	1,523	3,450	1,234
TCGA-AR-A1AY	6,755	2,616	5,400	2,295
TCGA-AR-A24Z	6,583	2,478	5,192	2,141
TCGA-AR-A256	19,993	9,103	18,465	8,645
TCGA-AR-A2LK	5,246	2,024	4,122	1,737
TCGA-B6-A0I1	8,138	3,179	6,752	2,854
TCGA-B6-A0WX	5,406	1,954	4,199	1,665
TCGA-BH-A0AV	9,292	3,944	7,960	3,652
TCGA-BH-A0BW	11,001	4,420	9,527	4,066
TCGA-BH-A0DG	4,859	1,730	3,710	1,483
TCGA-BH-A0DT	3,140	939	2,001	703
TCGA-BH-A0EA	4,055	1,330	2,921	1,095
TCGA-BH-A0H0	4,314	1,504	3,252	1,262
TCGA-BH-A0H6	4,256	1,464	3,070	1,215
TCGA-BH-A1FC	12,007	5,250	10,604	4,872
TCGA-D8-A27F	10,159	4,242	8,940	3,980
TCGA-D8-A27H	13,412	6,069	12,040	5,714
TCGA-E2-A109				

	10,939	4,571	9,586	4,215
TCGA-E2-A14X	5,194	1,892	4,050	1,642
TCGA-E2-A156	4,033	1,376	2,852	1,141
TCGA-E2-A15K	12,518	5,263	11,031	4,889
TCGA-E2-A1LG	16,200	7,317	14,893	6,969
TCGA-E2-A1LK	6,447	2,317	5,188	1,995
TCGA-E2-A1LL	10,990	4,840	9,728	4,551
TCGA-E9-A1NH	4,551	1,652	3,422	1,425
TCGA-EW-A1J5	45,235	21,536	43,263	20,895
TCGA-EW-A1P8	5,052	1,699	3,731	1,430
TCGA-EW-A1PB	17,210	7,848	15,840	7,504
TCGA-EW-A1PC	22,800	10,450	21,247	9,982
TCGA-EW-A1PH	7,997	3,291	6,587	2,914
TCGA-EW-A3U0	19,036	8,374	17,467	7,959
TCGA-GI-A2C9	16,053	7,072	14,662	6,693
TCGA-GM-A2DF	5,049	1,768	3,720	1,500
TCGA-GM-A3XL	10,877	4,760	9,592	4,472
Total	497,741	209,130	432,021	193,331
Mean	9,955	4,183	8,640	3,867
Standard deviation	7,024	3,439	6,892	3,370

The number of mutations is shown for each of the 50 TCGA replication samples. Similarly to the TCGA discovery samples (Supplementary Table 2), mutations within repeat elements and in the same loci of known SNPs were filtered out in order to lower the false-positive rate.

Supplementary Table 7: Aberrantly expressed targets of the ten putative driver DHSs

DHS	Gene	Expression	Role in cancer
chr15:78292337-78292883	*ACSBG1*	Downregulated	Aberrantly expressed in breast cancer[8]
chr1:21660551-21662029	*ALPL*	Downregulated	
chr20:62115827-62119284	*ARFGAP1*	Overexpressed	Involved in microsatellite instability oncogenesis[9]
chr1:928510-931954	*ATAD3B*	Overexpressed	Associated with breast cancer progression and prognosis[10]
chr1:928510-931954	*C1orf159*	Overexpressed	
chr5:1325957-1328153	*CLPTM1L*	Overexpressed	Associated with breast cancer risk[11-14]; 20 kb upstream of *TERT*
chr20:62115827-62119284	*COL20A1*	Overexpressed	Used in expression microarray breast cancer risk predictive model[15]
chr20:62115827-62119284	*DNAJC5*	Overexpressed	
chr1:928510-931954	*DVL1*	Overexpressed	Aberrantly expressed in testicular cancer[16]
chr1:21660551-21662029	*ECE1*	Downregulated	Involved in prostate cancer cell invasion[17]
chr2:216531968-216533440	*FN1*	Overexpressed	Associated with several cancer types[18,19]
chr20:62115827-62119284	*GMEB2*	Overexpressed	
chr1:185903622-185904645	*HMCN1*	Downregulated	Mutated in gastric and colorectal cancer[20]
chr15:78292337-78292883	*IREB2*	Downregulated	Involved in lung cancer[21]
chr1:928510-931954	*ISG15*	Overexpressed	Inhibits cancer cell growth and promotes apoptosis[22]
chr5:1325957-1328153	*LPCAT1*	Overexpressed	Upregulation in breast cancer is associated with tumor progression[23]
chr1:46476793-46477209	*MAST2*	Overexpressed	Translocation partner of *NOTCH1* in breast cancer[24]
chr1:928510-931954	*MXRA8*	Downregulated	
chr20:62115827-62119284	*NKAIN4*	Downregulated	
chr1:46476793-46477209	*RAD54L*	Overexpressed	Associated with breast cancer progression and prognosis[25]
chr20:62115827-62119284	*SLC2A4RG*	Overexpressed	
chr1:928510-931954	*TNFRSF4*	Overexpressed	Involved in head and neck squamous cell carcinoma[26]
chr6:28948439-28951450	*TRIM27*	Overexpressed	Known oncogene included in the Cancer Gene Census[27]
chr1:928510-931954	*TTLL10*	Downregulated	
chr1:928510-931954	*VWA1*	Overexpressed	Aberrantly methylated in ovarian cancer[28]
chr20:62115827-62119284	*YTHDF1*	Overexpressed	
chr8:579137-581436	*ZNF596*	Downregulated	Downregulated in breast cancer[29] and osteosarcoma[30]

For each of the 27 genes that are aberrantly expressed when the ten putative driver DHSs are mutated, their expression and role in cancer are shown.

Supplementary Table 8: Clusters analysis of the mutation rate in the ten driver DHSs by comparison to 20 random DHSs with similar genomic properties in all cancer types

DHS				20 random DHSs with similar properties			Tests		
**DHS**	**Number of mutations**	**Length (bp)**	**Mutation rate**	**Number of mutations**	**Length (bp)**	**Mutation rate**	**Log2 (odds ratio)**	**P-value**	**FDR < 0.05 threshold**
chr1:185903622-185904645	0	846	0.0000	14	16019	0.0009	-10.00	1.00E+00	1.30E-02
chr1:21660551-21662029	11	929	0.0118	39	6513	0.0060	0.98	2.71E-02	1.30E-02
chr1:46476793-46477209	3	400	0.0075	38	5306	0.0072	0.07	5.46E-01	1.30E-02
chr1:928510-931954	21	1721	0.0122	227	27883	0.0081	0.58	4.82E-02	1.30E-02
chr15:78292337-78292883	0	534	0.0000	3	3913	0.0008	-10.00	1.00E+00	1.30E-02
chr16:28081546-28081590	0	38	0.0000	0	4552	0.0000	0.00	N/A	1.30E-02
chr19:14191744-14193178	10	1028	0.0097	74	10395	0.0071	0.45	2.03E-01	1.30E-02
chr2:216531968-216533440	9	1389	0.0065	49	6950	0.0071	-0.12	6.43E-01	1.30E-02
chr2:218332114-218332448	2	283	0.0071	49	6950	0.0071	0.00	5.93E-01	1.30E-02
chr20:62115827-62119284	60	2679	0.0224	145	15400	0.0094	1.25	2.90E-09	1.30E-02
chr22:45987462-45987772	2	291	0.0069	46	6828	0.0067	0.03	5.83E-01	1.30E-02
chr3:196189710-196189944	0	222	0.0000	3	3656	0.0008	-10.00	1.00E+00	1.30E-02
chr5:102609590-102610013	0	404	0.0000	22	3803	0.0058	-10.00	1.00E+00	1.30E-02
chr5:1325957-1328153	73	2147	0.0340	272	36059	0.0075	2.17	4.44E-16	1.30E-02
chr6:28948439-28951450	16	1959	0.0082	32	5152	0.0062	0.40	1.68E-01	1.30E-02
chr8:579137-581436	105	2145	0.0490	44	4334	0.0102	2.27	5.55E-16	1.30E-02

From the clusters defined in Supplementary Data 4, for each of the ten driver DHSs 20 random DHSs with similar genomic properties (GC content, gene density in the surrounding 500-kb region, open chromatin, DNA replication time and expected mutations based on trinucleotide composition) were selected and their mutations were detected in all cancer types (Figure 3B). Log2 odds ratio was calculated between the mutation rate in the DHS and the mutation rate in its associated 20 random DHSs. The test statistic p was calculated assuming the Poisson model described for the analysis of breast cancer, with $\hat{\mu}$ for each driver DHS inferred from the set of 20 random DHSs. The significance threshold p^* was calculated as for Figure 2C (Eq. 4), requiring FDR < 0.05 ($p^* = 0.013$).

Supplementary Table 9: Description of the mutations validated in *C. intestinalis*

Mutation	Effect on TFBS	Mutated tumors	Expression in reference allele	Expression in alternative allele	Change between ref and alt
chr6:28949254A>C	Increased affinity of GATA	Multiple mutations in ovarian cancer (3 tumors)	25% a6.5 lineage, 68% Endoderm, 65% Secondary notochord	13% a6.5 lineage, 63% endoderm, 58% Secondary notochord	Yes (50% decreased expression in a6.5 lineage)
chr6:28950040C>T	Potentially changed affinity of ETS (mutation 3 bp from GGAA core)	Breast cancer	25% a6.5 lineage, 68% Endoderm, 65% Secondary notochord	22% a6.5 lineage, 67% Endoderm, 68.5 Secondary notochord	No
chr6:28950050G>A	Potentially changed affinity of ETS (mutation 2 bp from GGAA core)	Mutations in three tumor types (stomach, bladder, melanoma)	25% a6.5 lineage, 68% Endoderm, 65% Secondary notochord	17% a6.5 lineage, 54% Endoderm, 53% Secondary notochord	Yes (>20% decrease in all three tissues)
chr6:28950885A>G	Loss of GATA site	Bladder cancer	4% Epidermis, 84% b6.5 lineage, 83% a6.5 lineage, 43% Endoderm	30% Epidermis, 87% b6.5 lineage, 84% a6.5 lineage, 52% Endoderm	Yes (7X increase expression in epidermis)

For each of the four mutations for which experimental validation was attempted, we show the position, the substitution type, the effects on transcription factor binding, the tumor types where each mutation was found, and a summary of the observed effects (tests are shown in Supplementary Table 10).

Supplementary Table 10: Effects of somatic mutations on *C. intestinalis* embryos

A) Replicates information

Construct	chr6:28,948,460-28,950,283							
Mutation	Wild-type		chr6:28949254A>C		chr6:28950040C>T		chr6:28950050G>A	
Lineage	Replicate 1	Replicate 2	Replicate 1	Replicate 2	Replicate 1	Replicate 2	Replicate 1	Replicate 2
b6.5 lineage	NA	NA	NA	NA	NA	NA	NA	NA
a6.5 lineage	22	28	9	17	26	17	19	15
Endoderm	60	75	50	75	66	68	54	54
Epidermis	NA	NA	NA	NA	NA	NA	NA	NA
Secondary notochord	61	69	53	63	73	64	55	50
Total tested embryos	100	100	100	100	100	100	100	100

Construct	chr6:28,950,315-28,951,026					
Mutation	Wild-type			chr6:28950885A>G		
Lineage	Replicate 1	Replicate 2	Replicate 3	Replicate 1	Replicate 2	Replicate 3
b6.5 lineage	39	49	84	45	42	83
a6.5 lineage	43	50	78	45	37	82
Endoderm	24	33	32	20	22	59
Epidermis	0	0	9	16	17	26
Secondary notochord	NA	NA	NA	NA	NA	NA
Total tested embryos	50	55	100	50	45	100

B) Tests to assess differences between wild-type and mutated enhancer

Mutations	Lineage	Mutated			Wild-type			P-value
		Expressed	Not expressed	% Expressed	Expressed	Not expressed	% Expressed	
chr6:28950040C>T	a6.5 lineage	43	157	21.5	50	150	25.0	4.78E-01
chr6:28950050G>A	a6.5 lineage	34	166	17.0	50	150	25.0	6.52E-02
chr6:28949254A>C	a6.5 lineage	26	174	13.0	50	150	25.0	3.20E-03
chr6:28950040C>T	Endoderm	134	66	67.0	135	65	67.5	1.00E+00
chr6:28950050G>A	Endoderm	108	92	54.0	135	65	67.5	7.67E-03
chr6:28949254A>C	Endoderm	125	75	62.5	135	65	67.5	3.45E-01
chr6:28950040C>T	Secondary notochord	137	63	68.5	130	70	65.0	5.24E-01

chr6:28950050G>A	Secondary notochord	105	95	52.5	130	70	65.0	1.47E-02
chr6:28949254A>C	Secondary notochord	116	84	58.0	130	70	65.0	1.82E-01
chr6:28950885A>G	b6.5 lineage	170	25	87.2	172	33	83.9	3.95E-01
chr6:28950885A>G	a6.5 lineage	164	31	84.1	171	34	83.4	8.93E-01
chr6:28950885A>G	Endoderm	101	94	51.8	89	116	43.4	1.09E-01
chr6:28950885A>G	Epidermis	59	136	30.3	9	196	4.4	1.25E-12

Shown are the lineages where GFP expression associated with each mutation was assessed. (**A**) Two or three replicates were conducted for each mutation test. For each replicate, the number of embryos that express the enhancer are shown. Two regions were tested with one wild type construct used per region. One of the two regions had three mutations examined. (**B**) The number of expressed and not expressed embryos, as well as the percentage of embryos showing GFP expression, is shown for the mutated and wild-type embryos combined across all replicates. P-values from Fisher's exact test are shown.

Supplementary Table 11: Primers used for experimental validation *in vivo*

Location	Mutation	Size (bp)	Fwd Primer	Rev Primer	Fwd Mutagenesis Primer	Rev Mutagenesis Primer
chr6:28,950,315-28,951,026	chr6:28950885A>G	712	CATCATGGCGCGCCTTGGGTGATTCAGTAAGCGG	CATCATTCTAGATAAGATCAGGAGCACCACGA	GTCTGACAATGTCGGGGCATGAATCTTTGTTTCTT	CATGCCCCGACATTGTCAGACAGGAACCAGTTAGC
chr6:28,948,460-28,950,283	chr6:28949254A>C	1,824	CATCATGGCGCGCCCGAGTAGGAAGACAGGGGTTG	CATCATTCTAGAAACAATGCCGGACACTCGGT	CTTTTCTTCTGTTATCTAGTGAGATGTTGAAACCC	CACTAGATAACAGAAGAAAAGGCAAACGTGCTGAA
chr6:28,948,460-28,950,283	chr6:28950050G>A	1,824	CATCATGGCGCGCCCGAGTAGGAAGACAGGGGTTG	CATCATTCTAGAAACAATGCCGGACACTCGGT	TCGATTCCCGATCAGGGAATGAGGTTTTTCTGTTT	CATTCCCTGATCGGGAATCGAACCCGGGCCGCGGC
chr6:28,948,460-28,950,283	chr6:28950040C>T	1,824	CATCATGGCGCGCCCGAGTAGGAAGACAGGGGTTG	CATCATTCTAGAAACAATGCCGGACACTCGGT	GTCAGGGAATAAGGTTTTTCTGTTTTAACCTCCAA	AGAAAAACCTTATTCCCTGACCGGGAATCGAACCC

List of primers used for the *C. intestinalis* experiment.

Supplementary Table 12: sgRNAs used for CRISPR

Name	Sequence 5' to 3'
chr8:579138_T_5'_1 F	**CACC** **G** CTG AGC ACA CGT GAT CAT A
chr8:579138_T_5'_1 R	**AAAC** T ATG ATC ACG TGT GCT CAG **C**
chr8:579138_T_3'_1 F	**CACC** GCG CAG ACA TCG GAC TGT TA
chr8:579138_T_3'_1 R	**AAAC** TA ACA GTC CGA TGT CTG CGC
chr20-62117780 - T-5'-1 F	**CACC** GCC TCG CCA TTT GCC GTC AT
chr20-62117780 - T-5'-1 R	**AAAC** AT GAC GGC AAA TGG CGA GGC
chr20-62117780 - T-3'-1 F	**CACC** GTT CCT ACA ACC ACG TAC AG
chr20-62117780 - T-3'-1 R	**AAAC** CT GTA CGT GGT TGT AGG AAC

The table provides the sequences of the sgRNAs used to delete the putative driver DHSs with CRISPR. The sequences highlighted in red were added to clone the sgRNAs into ph174 vectors.

Supplementary Table 13: PCR primers to confirm the deletion of putative driver DHS with CRISPR

Name	Sequence 5' to 3'
chr8_579138_T_1 F	CATAAAGACTAAAGGAGGTGG
chr8_579138_T_2 R	GAGGAGAGACCGAAATTCTCA
chr8_579138_T_6 F	CATGGCCTGAAGCTTGGCTC
chr20_62117780_T_1 F	TCGTTTCCGTCCTCACCTAG
chr20_62117780_T_2 F	CATCACCACTGCCTGTCACCTG
chr20_62117780_T_1R	CACTCTCCTGCTAAGCCGGTC
chr20_62117780_T_3 R	GTTCCCTGAGGATGCAGGTG

List of primers used for PCRs to confirm the deletion of the putative driver DHSs and the control regions.

Supplementary Table 14: Metadata for the samples that underwent ATAC-seq and RNA-seq

Sample ID	Driver DHS tested	Deleted interval	Condition	Clone ID	Harvest date	Use
emptyvector_1	chr8:579137-581436	NA	emptyvector	chr8_PHI74_4	12/3/2014	ATAC-seq + RNA-seq
emptyvector_2	chr8:579137-581436	NA	emptyvector	chr8_PHI74_3	10/27/2014	RNA-seq
emptyvector_3	chr8:579137-581436	NA	emptyvector	chr8_PHI74_2	10/1/2014	RNA-seq
dhs_1	chr8:579137-581436	chr8:579149-581504	deleted driver DHS	chr8_T_43	10/23/2014	RNA-seq
dhs_2	chr8:579137-581436	chr8:579149-581504	deleted driver DHS	chr8_T_44	10/23/2014	RNA-seq
dhs_3	chr8:579137-581436	chr8:579149-581504	deleted driver DHS	chr8_T_51	12/5/2014	RNA-seq
dhs_4	chr8:579137-581436	chr8:579149-581504	deleted driver DHS	chr8_T_58	12/5/2014	ATAC-seq + RNA-seq
emptyvector_5	chr20:62115827-62119284	NA	emptyvector	chr20_PHI74_1	11/22/2014	RNA-seq
emptyvector_6	chr20:62115827-62119284	NA	emptyvector	chr20_PHI74_2	11/24/2014	RNA-seq
emptyvector_7	chr20:62115827-62119284	NA	emptyvector	chr20_PHI74_3	11/26/2014	RNA-seq
emptyvector_8	chr20:62115827-62119284	NA	emptyvector	chr20_PHI74_4	11/30/2014	RNA-seq
dhs_5	chr20:62115827-62119284	chr20:62117977-62118878	deleted driver DHS	chr20_T_10	11/26/2014	RNA-seq
dhs_6	chr20:62115827-62119284	chr20:62117977-62118878	deleted driver DHS	chr20_T_36	12/1/2014	RNA-seq
dhs_7	chr20:62115827-62119284	chr20:62117977-62118878	deleted driver DHS	chr20_T_43	12/1/2014	RNA-seq
dhs_8	chr20:62115827-62119284	chr20:62117977-62118878	deleted driver DHS	chr20_T_46	12/2/2014	RNA-seq
dhs_9	chr20:62115827-62119284	chr20:62117977-62118878	deleted driver DHS	chr20_T_9	11/28/2014	RNA-seq

Metadata for the samples that underwent ATAC-seq and RNA-seq. Samples with the same clone ID are technical replicates. Harvest date refers to the date when samples were processed and pellets were frozen. All samples were processed simultaneously for DNA and RNA extraction (ATAC-seq and RNA-seq), library generation and sequencing.

Supplementary References

1 Gradishar, W. J. *et al.* Breast cancer version 3.2014. *Journal of the National Comprehensive Cancer Network : JNCCN* **12**, 542-590 (2014).

2 Banerji, S. *et al.* Sequence analysis of mutations and translocations across breast cancer subtypes. *Nature* **486**, 405-409, doi:nature11154 (2012).

3 Network, T. C. G. A. R. Comprehensive molecular portraits of human breast tumours. *Nature* **490**, 61-70, doi:nature11412 (2012).

4 Stephens, P. J. *et al.* The landscape of cancer genes and mutational processes in breast cancer. *Nature* **486**, 400-404, doi:nature11017 (2012).

5 Tamborero, D. *et al.* Comprehensive identification of mutational cancer driver genes across 12 tumor types. *Scientific reports* **3**, 2650, doi:10.1038/srep02650 (2013).

6 Wilks, C. *et al.* The Cancer Genomics Hub (CGHub): overcoming cancer through the power of torrential data. *Database : the journal of biological databases and curation* **2014**, doi:10.1093/database/bau093 (2014).

7 Yost, S. E. *et al.* Mutascope: sensitive detection of somatic mutations from deep amplicon sequencing. *Bioinformatics* **29**, 1908-1909, doi:10.1093/bioinformatics/btt305 (2013).

8 Makoukji, J. *et al.* Gene expression profiling of breast cancer in Lebanese women. *Scientific reports* **6**, 36639, doi:10.1038/srep36639 (2016).

9 Sangar, F. *et al.* Involvement of small ArfGAP1 (SMAP1), a novel Arf6-specific GTPase-activating protein, in microsatellite instability oncogenesis. *Oncogene* **33**, 2758-2767, doi:10.1038/onc.2013.211 (2014).

10 Ovaska, K. *et al.* Integrative analysis of deep sequencing data identifies estrogen receptor early response genes and links ATAD3B to poor survival in breast cancer. *PLoS computational biology* **9**, e1003100, doi:10.1371/journal.pcbi.1003100 (2013).

11 Liu, C. L. *et al.* Association between CLPTM1L-TERT rs401681 polymorphism and risk of pancreatic cancer: a meta-analysis. *Clinical and experimental medicine*, doi:10.1007/s10238-014-0316-3 (2014).

12 Yin, Z. *et al.* Genetic polymorphisms of TERT and CLPTM1L, cooking oil fume exposure, and risk of lung cancer: a case-control study in a Chinese non-smoking female population. *Medical oncology* **31**, 114, doi:10.1007/s12032-014-0114-5 (2014).

13 Zhang, Y. *et al.* Genetic polymorphisms of TERT and CLPTM1L and risk of lung cancer: a case-control study in northeast Chinese male population. *Medical oncology* **31**, 18, doi:10.1007/s12032-014-0018-4 (2014).

14 Zhao, D. P., Yang, C. L., Zhou, X., Ding, J. A. & Jiang, G. N. Association between CLPTM1L polymorphisms (rs402710 and rs401681) and lung cancer susceptibility: evidence from 27 case-control studies. *Molecular genetics and genomics : MGG* **289**, 1001-1012, doi:10.1007/s00438-014-0868-7 (2014).

15 Huang, C. C. *et al.* Concurrent gene signatures for han chinese breast cancers. *PloS one* **8**, e76421, doi:10.1371/journal.pone.0076421 (2013).

16 Fabijanovic, D. *et al.* The expression of SFRP1, SFRP3, DVL1, and DVL2 proteins in testicular germ cell tumors. *APMIS : acta pathologica, microbiologica, et immunologica Scandinavica* **124**, 942-949, doi:10.1111/apm.12588 (2016).

17 Whyteside, A. R., Hinsley, E. E., Lambert, L. A., McDermott, P. J. & Turner, A. J. ECE-1 influences prostate cancer cell invasion via ET-1-mediated FAK phosphorylation and ET-1-independent mechanisms. *Canadian journal of physiology and pharmacology* **88**, 850-854, doi:10.1139/Y10-054 (2010).

18 Lee, J. C. *et al.* Characterization of FN1-FGFR1 and novel FN1-FGF1 fusion genes in a large series of phosphaturic mesenchymal tumors. *Modern pathology : an official journal of the United States and Canadian Academy of Pathology, Inc*, doi:10.1038/modpathol.2016.137 (2016).

19 Panagopoulos, I. *et al.* Recurrent fusion of the genes FN1 and ALK in gastrointestinal leiomyomas. *Modern pathology : an official journal of the United States and Canadian Academy of Pathology, Inc*, doi:10.1038/modpathol.2016.129 (2016).

20 Lee, S. H., Je, E. M., Yoo, N. J. & Lee, S. H. HMCN1, a cell polarity-related gene, is somatically mutated in gastric and colorectal cancers. *Pathology oncology research : POR* **21**, 847-848, doi:10.1007/s12253-014-9809-3 (2015).

21 Liu, Y. *et al.* Focused Analysis of Exome Sequencing Data for Rare Germline Mutations in Familial and Sporadic Lung Cancer. *Journal of thoracic oncology : official publication of the International Association for the Study of Lung Cancer* **11**, 52-61, doi:10.1016/j.jtho.2015.09.015 (2016).

22 Zhou, M. J. *et al.* ISG15 inhibits cancer cell growth and promotes apoptosis. *International journal of molecular medicine* **39**, 446-452, doi:10.3892/ijmm.2016.2845 (2017).

23 Abdelzaher, E. & Mostafa, M. F. Lysophosphatidylcholine acyltransferase 1 (LPCAT1) upregulation in breast carcinoma contributes to tumor progression and predicts early tumor recurrence. *Tumour biology : the journal of the International Society for Oncodevelopmental Biology and Medicine*, doi:10.1007/s13277-015-3214-8 (2015).

24 Clay, M. R., Varma, S. & West, R. B. MAST2 and NOTCH1 translocations in breast carcinoma and associated pre-invasive lesions. *Human pathology* **44**, 2837-2844, doi:10.1016/j.humpath.2013.08.001 (2013).

25 Gee, H. E. *et al.* MicroRNA-Related DNA Repair/Cell-Cycle Genes Independently Associated With Relapse After Radiation Therapy for Early Breast Cancer. *International journal of radiation oncology, biology, physics* **93**, 1104-1114, doi:10.1016/j.ijrobp.2015.08.046 (2015).

26 Bell, R. B. *et al.* OX40 signaling in head and neck squamous cell carcinoma: Overcoming immunosuppression in the tumor microenvironment. *Oral oncology* **52**, 1-10, doi:10.1016/j.oraloncology.2015.11.009 (2016).

27 Futreal, P. A. *et al.* A census of human cancer genes. *Nature reviews. Cancer* **4**, 177-183, doi:10.1038/nrc1299 (2004).

28 Cicek, M. S. *et al.* Epigenome-wide ovarian cancer analysis identifies a methylation profile differentiating clear-cell histology with epigenetic silencing of the HERG K+ channel. *Human molecular genetics* **22**, 3038-3047, doi:10.1093/hmg/ddt160 (2013).

29 Finak, G. *et al.* Stromal gene expression predicts clinical outcome in breast cancer. *Nature medicine* **14**, 518-527, doi:10.1038/nm1764 (2008).

30 Bruheim, S., Xi, Y., Ju, J. & Fodstad, O. Gene expression profiles classify human osteosarcoma xenografts according to sensitivity to doxorubicin, cisplatin, and ifosfamide. *Clinical cancer research : an official journal of the American Association for Cancer Research* **15**, 7161-7169, doi:10.1158/1078-0432.CCR-08-2816 (2009).
